# Structural tissue damage and 24-month progression of semi-quantitative MRI biomarkers of knee osteoarthritis in the IMI-APPROACH cohort

**DOI:** 10.1186/s12891-022-05926-1

**Published:** 2022-11-17

**Authors:** Frank W. Roemer, Mylène Jansen, Anne C. A. Marijnissen, Ali Guermazi, Rafael Heiss, Susanne Maschek, Agnes Lalande, Francisco J. Blanco, Francis Berenbaum, Lotte A. van de Stadt, Margreet Kloppenburg, Ida K. Haugen, Christoph H. Ladel, Jaume Bacardit, Anna Wisser, Felix Eckstein, Floris P. J. G. Lafeber, Harrie H. Weinans, Wolfgang Wirth

**Affiliations:** 1 Department of Radiology, Universitätsklinikum Erlangen and Friedrich-Alexander-Universität Erlangen-Nürnberg (FAU), Maximiliansplatz 3, 91054 Erlangen, Germany; 2 Quantitative Imaging Center, Department of Radiology, Boston University School of Medicine, Boston, MA USA; 3 University Medical Center Utrecht, Utrecht, The Netherlands; 4 Department of Radiology, VA Boston Healthcare System, West Roxbury, MA USA; 5 Chondrometrics GmbH, Freilassing, Germany; 6 Department of Imaging & Functional Musculoskeletal Research, Institute of Anatomy & Cell Biology, Paracelsus Medical University Salzburg & Nuremberg, Salzburg, Austria; 7 Institut de Recherches Internationales Servier, Suresnes, France; 8 Servicio de Reumatologia, INIBIC- Universidade de A Coruña, A Coruña, Spain; 9 Department of Rheumatology, AP-HP Saint-Antoine Hospital, Paris, France; 10 INSERM, Sorbonne University, Paris, France; 11 Department of Rheumatology, Leiden University Medical Center, Leiden, The Netherlands; 12 Clinical Epidemiology, Leiden University Medical Center, Leiden, The Netherlands; 13 Division of Rheumatology and Research, Diakonhjemmet Hospital, Oslo, Norway; 14 Independent Consultant, Darmstadt, Germany; 15 School of Computing, Newcastle University, Newcastle, UK; 16 Ludwig Boltzmann Inst. for Arthritis and Rehabilitation, Paracelsus Medical University Salzburg & Nuremberg, Salzburg, Austria

**Keywords:** Osteoarthritis, Knee, MRI, Progression; reliability

## Abstract

**Background:**

The IMI-APPROACH cohort is an exploratory, 5-centre, 2-year prospective follow-up study of knee osteoarthritis (OA). Aim was to describe baseline multi-tissue semiquantitative MRI evaluation of index knees and to describe change for different MRI features based on number of subregion-approaches and change in maximum grades over a 24-month period.

**Methods:**

MRIs were acquired using 1.5 T or 3 T MRI systems and assessed using the semi-quantitative MRI OA Knee Scoring (MOAKS) system. MRIs were read at baseline and 24-months for cartilage damage, bone marrow lesions (BML), osteophytes, meniscal damage and extrusion, and Hoffa- and effusion-synovitis. In descriptive fashion, the frequencies of MRI features at baseline and change in these imaging biomarkers over time are presented for the entire sample in a subregional and maximum score approach for most features. Differences between knees without and with structural radiographic (R) OA are analyzed in addition.

**Results:**

Two hundred eighty-nine participants had readable baseline MRI examinations. Mean age was 66.6 ± 7.1 years and participants had a mean BMI of 28.1 ± 5.3 kg/m^2^. The majority (55.3%) of included knees had radiographic OA. Any change in total cartilage MOAKS score was observed in 53.1% considering full-grade changes only, and in 73.9% including full-grade and within-grade changes. Any medial cartilage progression was seen in 23.9% and any lateral progression on 22.1%. While for the medial and lateral compartments numbers of subregions with improvement and worsening of BMLs were very similar, for the PFJ more improvement was observed compared to worsening (15.5% vs. 9.0%). Including within grade changes, the number of knees showing BML worsening increased from 42.2% to 55.6%. While for some features 24-months change was rare, frequency of change was much more common in knees with vs. without ROA (e.g. worsening of total MOAKS score cartilage in 68.4% of ROA knees vs. 36.7% of no-ROA knees, and 60.7% vs. 21.8% for an increase in maximum BML score per knee).

**Conclusions:**

A wide range of MRI-detected structural pathologies was present in the IMI-APPROACH cohort. Baseline prevalence and change of features was substantially more common in the ROA subgroup compared to the knees without ROA.

**Trial Registration:**

Clinicaltrials.gov identification: NCT03883568.

**Supplementary Information:**

The online version contains supplementary material available at 10.1186/s12891-022-05926-1.

## Background

Osteoarthritis (OA) is a highly prevalent chronic condition with marked implications for affected individuals and public health care [1, 2]. Current treatment approaches focus on controlling symptoms since there are no interventions that have yet been approved for modifying the course of the disease or improving structural alterations in affected joint tissues [3]. Non-pharmacological and non-surgical methods such as education and self-management, exercise, weight loss if overweight or obese, and walking aids as indicated, are widely recommended and seen as first-line treatment [4].

OA is understood today as the clinical and pathologic outcome of a range of disorders that result in structural and functional failure of synovial joints. Joint imaging, particularly magnetic resonance imaging (MRI), has evolved rapidly in recent years due to technical advances and their application to clinical research, which has led to abundant evidence regarding the natural history of the disease [5]. While radiography depicts structural bony tissue changes only in advanced stages of OA, MRI is able to visualize all involved joint tissues, even in the earliest stages of disease when radiographs appear still normal [6]. Recent data suggest that non-cartilaginous tissue changes in particular play an important role in the onset and progression of OA [7, 8]. MRI-based semi-quantitative (SQ) scoring of knee OA is a valuable method for performing multi-tissue joint assessment in observational cross-sectional and longitudinal studies of OA including clinical trials [9]. SQ scoring enables evaluation of the whole knee joint using MRI acquisition techniques that are commonly applied in a clinical environment [10]. SQ scoring has expanded our understanding of disease onset and progression and plays an increasing role regarding clinical trial design [11, 12].

In the Foundation for the National Institutes of Health (FNIH) Osteoarthritis Biomarkers Consortium study,—a nested case–control study based within the larger Osteoarthritis Initiative (OAI) study -, presence and amount of baseline structural tissue damage and worsening of several MRI features from baseline to 24 months were associated with increased odds of progression as defined by pre-determined radiographic, clinical or combined outcomes [8].

The IMI-APPROACH (Innovative Medicines Initiative—Applied Public–Private Research enabling OsteoArthritis Clinical Headway, https://www.approachproject.eu) study is an exploratory, European, 5-centre, 2-year prospective follow-up cohort project [13]. Although currently available cohort studies, like the Dutch CHECK [14] and the US OAI with the FNIH subcohort [8] have increased our knowledge of the disease, these attempts still have not resulted in clearly distinctive phenotypes/endotypes with predictive biomarkers. IMI-APPROACH was designed to prospectively describe pre-identified progressor phenotypes of patients with symptomatic and/or structural knee OA by use of conventional and novel clinical, imaging, and biochemical biomarkers, and to validate and refine a predictive model for progressor phenotypes based on these markers. The recruitment for IMI-APPROACH was based on rankings produced by machine-learning models that were trained using data from existing cohorts to estimate the likelihood of joint space width loss (so-called s-score) and/or increased or sustained knee pain (p-score) over the course of the study from demographic data, pain scores, and radiographic features [13, 15]. In addition to this unique selection of participants, the IMI-APPROACH cohort combines a broad spectrum of conventional and novel, explorative, imaging, biochemical, clinical and demographic markers. Modern data science techniques suitable to analyze such extensive datasets will help identifying and predicting phenotypes/endotypes of OA that share distinct underlying pathobiological mechanisms with their structural and functional consequences, relevant for clinical practice and targeted clinical trials.

Herewith, we describe the scoring methodology and baseline cross-sectional frequencies of structural joint tissue damage in IMI-APPROACH participants based on SQ MRI assessment. Furthermore, we describe the extent of changes over a 24-month period for the different MRI features in the overall sample and compare subgroups with and without radiographic knee OA, which may serve as a potential reference for future studies focusing on MRI features and progression over similar observational periods. Finally, we report cross-sectional and longitudinal reliability of MRI assessments,

## Methods

### Study characteristics

IMI-APPROACH is an observational, longitudinal study that enrolled 297 OA patients at five clinical centers in Europe [13, 15]. The study participants were recruited from five existing observational OA cohorts (CHECK (Utrecht, The Netherlands) [14], HOSTAS (Leiden, The Netherlands) [16], MUST (Oslo, Norway) [17], PROCCOAC (A Coruña, Spain) [18], and DIGICOD (Paris, France) [19]) or from outpatient departments, if not enough participants could be recruited from these existing cohorts. Recruitment from these cohorts relied on machine-learning models that were trained using data from the CHECK cohort and the OAI to predict either the probability of increased or sustained knee pain or the probability of structural progression over the next 2 years. These models were then applied to X-ray-based measures, demographic and clinical data collected at the screening visit to select OA patients with the highest likelihood of having pain and/or structural progression over the course of the study [20]. Beyond the machine learning-based rankings, additional inclusion criteria were ability to walk unassisted, predominantly tibiofemoral knee OA and satisfying the clinical America College of Rheumatology (ACR) classification criteria for knee OA; exclusion criteria included participation in a trial of local therapeutic intervention for index knee OA or potential systemic disease modifying OA drugs (DMOADs) at the time of inclusion, within six months before inclusion, and/or anticipated during two years of follow-up, surgery of the index knee in the six months before inclusion and/or scheduled or expected surgery of the index knee during follow-up, current pregnancy or planned pregnancy during follow-up, predominantly patellofemoral knee OA and others such as alternative/additional causes of joint pain, for example, rheumatic symptoms due to malignancies, primary osteochondromatosis or osteonecrosis [13]. After inclusion and exclusion criteria were checked an index knee was selected based on ACR criteria. If both knees fulfilled the criteria, patients indicated their own index knee based on severity of complaints, in case equal the right knee was selected as the index knee. In case both knees were affected equally, the right knee was selected as the index knee. Demographic and clinical data, blood and urine samples, and imaging data were collected. Regarding imaging, X-ray of the index knee at screening, 6, 12, and 24 months (X-ray of the contralateral knee only at enrolment and 24 month), MRI of the index knee at enrollment, month 6, 12, and 24 follow-up visits, and a CT of the index knee (to extract bone shape, bone mineral density and texture parameters of subchondral bone architecture; only at enrolment and 24 month) were acquired.

IMI-APPROACH was conducted in compliance with the protocol, Good Clinical Practice (GCP), the Declaration of Helsinki, and the applicable ethical and legal regulatory requirements (for all countries involved), and is registered under clinicaltrials.gov identifier: NCT03883568 (first submitted date 21/03/2019). All participants have received oral and written information and provided written informed consent.

#### MRI acquisition and evaluation

MRI of the index knee was acquired at the five clinical centers with two of the centers using 1.5 T systems (A Coruña: Ingenia CX, Philips Medical Systems, Netherlands; Oslo: Aera, Siemens Healthcare, Germany), and the other centers using 3 T systems (Utrecht: Ingenia or Achieva, Philips Medical Systems, Netherlands; Leiden: Ingenia, Philips Medical Systems, Netherlands; Paris: Skyra, Siemens Healthcare, Germany). The clinical pulse sequence protocol included an axial, a sagittal, a coronal intermediate-weighted fat-suppressed sequence and a T1-weighted coronal turbo spin echo sequence that were all used for SQ evaluation. Details of the pulse sequence protocol are presented in Appendix 1. In addition, a sagittal 3D spoiled gradient echo or volume-interpolated gradient echo sequence with selective water excitation or fat-suppression was acquired for the quantitative cartilage analysis. This sequence was also used for SQ assessment. After site qualification an inter-site comparison was performed with three volunteers who had both knees imaged at 4 of the 5 sites (1.5 T MRI: A Coruña and Oslo; 3.0 T: Leiden and Utrecht). For the analysis of test–retest precision, each site asked study participants at the baseline visit whether they volunteered into one additional MRI acquisition performed at both the baseline and the month 24 visit. Altogether 26 test–retest MRIs were acquired with repositioning of the knee between scans (patients were allowed but not required to leave the scanner) [21].

One musculoskeletal radiologist (FWR) with 17 years’ experience of SQ assessment of knee OA at the time of reading, blinded to all clinical data and predicted progressor status, read the MRIs according to the MRI Osteoarthritis Knee Score (MOAKS) instrument [22] with knowledge of the chronological order of the scans. The following joint structures were assessed: cartilage morphology, subchondral bone marrow lesions (BMLs), osteophytes, meniscal structural damage and meniscal extrusion, Hoffa-synovitis and effusion-synovitis. For the current study only the baseline and 24-months MRIs were considered.

In addition, within-grade changes were coded that fulfill the definition of a definite visual change but do not fulfill the definition of a full-grade change on the ordinal scales applied [23]. Within-grade changes were applied for cartilage and BML assessment. For cartilage, within-grade changes were coded for the area-extent dimension and the full-thickness dimension of the MOAKS scale, separately.

### Reliability

For reliability assessment, 20 MRIs were randomly selected to represent the spectrum of study sites and disease severity. Four knees were chosen from each site. The knees represented the spectrum of baseline disease severity from Kellgren-Lawrence (KL) grade 0 to 4 (four knees for each KL grade). The MRIs were assessed for the purpose of intra-reader reliability by the primary reader (FWR) in random order four weeks after the last study participant completed the MRI acquisitions and at least 6 weeks after the primary readings had been performed. Reliability assessment was performed in chronological order with time point known to the reader for baseline and 24-months including within-grade changes. A second reader with 19 years of experience of SQ MRI assessment of knee OA at the time of reading (AG) read baseline and 24-months follow-up MRI in identical fashion for assessment of inter-observer reliability.

### Features assessed and change over time

**Fig. 1:**
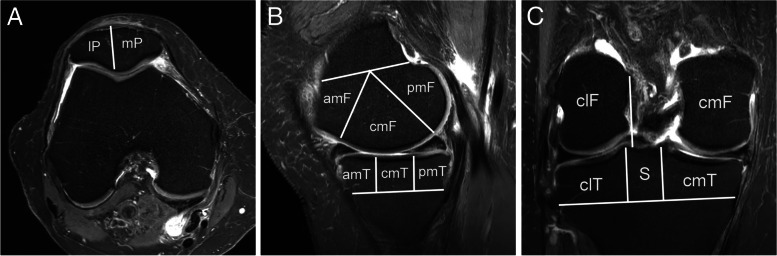
Subregional division for cartilage and bone marrow lesion assessment using the MOAKS instrument. Both features are assessed in 14 articular subregions. **A**. Axial intermediate-weighted fat suppressed image shows subregional division of the patella into the medial (mP) and lateral patella (lP). Note that the patella apex is part of the medial patella. **B**. Sagittal intermediate-weighted fat suppressed image of the medial compartment shows the three femoral and three tibial subregions. The femur is subdivided into the anterior (amF), central (cmF) and posterior (pmF) subregions. The tibia is subdivided into the anterior (amT), central (cmT) and posterior (pmT) subregions. The lateral compartment is subdivided in corresponding fashion in the sagittal plane (not shown). **C**. Coronal intermediate-weighted fat suppressed image shows the central femoral and tibial subregions. The tibial S region (subspinous – adjacent to the tibial spines) is not considered for BML and cartilage evaluation

*Cartilage ―* MOAKS uses a two-digit score for cartilage assessment (each 0–3) that incorporates both area size per subregion (i.e. in the following referred to as “area extent”-dimension) and percentage of subregion that is affected by full-thickness cartilage loss (i.e. in this analysis referred to as “full-thickness”-dimension). Figure 1 depicts the subregional division for cartilage (and BML) assessment for the femur, tibia and the patella. Frequencies are presented for maximum MOAKS score and number of subregions affected by any cartilage damage on a knee level. In addition, the area extent and full-thickness results are presented separately for the whole knee and on a compartmental level. The number of subregions with worsening (i.e., a higher score at 24 months vs. baseline) was defined for the total MOAKS score and separately for area extent and full-thickness. Change over time on a knee and compartmental level was defined as increase in number of subregions showing any MOAKS cartilage worsening including within-grade changes and excluding-within grade changes. Within-grade scoring for cartilage refers to any within-grade change in area or thickness for the total MOAKS score evaluations, and considered separately for area extent and full-thickness dimensions. In addition, change was categorized into none vs. any change.

*BMLs ―* MOAKS assesses BMLs in three dimensions: % of subregion affected by any (ill-defined and/or cystic) BML (0–3), % of subregion that is cystic vs. ill-defined BML (0–3) and number of BMLs per subregion. Here we report only the size component as this aspect of BML assessment incorporates both the ill-defined and cystic part of lesion and is clinically most relevant [24]. Subchondral cysts are only marginally associated with symptoms [25]. BMLs are assessed in the same 14 articular subregions as cartilage with the exception that the tibial subspinous subregion is assessed in addition for BMLs. That subregion, however, was not considered as not covered by cartilage and lesions in this region are not considered subchondral. Number of subregions affected by any BML and maximum BML score are presented on a knee and compartmental level. Change in overall number of subregions affected by any BML was defined as the difference between the number of subregions affected by any BML at 24 months (size > 0) and the number of subregions affected by any BML at baseline. This was further categorized into improvement, no change, and worsening in one subregion and worsening in two or more subregions. Further, the maximum increase in BML score from baseline to 24 months was determined on a knee and compartmental level. Finally, the number of subregions with worsening, and the number of subregions with improvement was determined for full-grade changes only and for full-grade and within-grade changes combined. We classified these measures into any subregions with worsening and any subregions with improvement on a knee and compartmental level.

*Osteophytes ―* MOAKS assesses osteophytes at 12 possible marginal locations of the joint on a scale from 0 to 3. For baseline, number of locations with any osteophytes and the maximum osteophyte score are described for the knee and compartmental level. The change in number of locations affected by any osteophyte was defined as the difference between the number of locations affected by any osteophyte at 24 months (Grade > 0) and the number of locations affected by any osteophyte at baseline. This change was classified as no change, or any worsening, and for the numbers of locations affected by change. In addition, change in maximum osteophyte score, was defined as the greatest amount of worsening of all affected locations per knee or compartment. This was further dichotomized into any vs.no change in maximum score.

*Meniscus ―* MOAKS scores meniscus damage from 0 to 8 with grade 1 representing intrameniscal signal but no tear or maceration. Grades 2–5 represent different tear types and grades 6–8 reflect maceration, i.e. meniscal substance loss. In addition, meniscal root tears are considered separately as these are considered detrimental for joint health [26, 27]. Furthermore, meniscal extrusion was scored in the anterior and mid-joint locations from 0–3. We assessed whether there was worsening in meniscal morphology from baseline to 24 months in each of the three medial or lateral meniscal subregions. These were evaluated separately. We defined worsening as an increase in grade in at least one subregion. We further categorized worsening in meniscal morphology into number of subregions with any worsening and categorical change (i.e. from normal to tear, normal to maceration or tear to maceration). We assessed changes in meniscal extrusion and root tears separately in the medial and lateral compartments as any change vs. no change.

*Hoffa-Synovitis and Effusion-synovitis ―* As MRI markers of inflammation, so-called effusion- and Hoffa-synovitis are evaluated in MOAKS. Hoffa-synovitis is a term used for signal changes in Hoffa’s fat pad that are commonly used as a surrogate for synovitis on non-contrast-enhanced MRI [22]. Effusion-synovitis is scored from 0 to 3 according to the distention of the joint capsule as 1 = small, 2 = moderate and 3 = large. Hoffa-synovitis is scored based on the amount of hyperintensity signal in Hoffa’s fat pad on sagittal fat suppressed intermediate-weighted sequences as 1 = mild, 2 = moderate and 3 = severe. Frequencies of baseline Hoffa- and effusion synovitis are presented. 24-months changes in Hoffa-synovitis and effusion-synovitis are assessed separately and categorized as improvement, no change, or worsening.

*Analytic approach ―* Descriptive statistics are used to report frequencies for the different features and parameters for baseline and change over time. Data is presented for the entire sample and for those knees with and those without radiographic OA. Mann–Whitney-U test was applied to describe differences between knees without radiographic OA (i.e. KL 0 and 1) vs. those with radiographic OA (i.e. KL 2–4). For some features raw distributions were grouped into categories as described above. In these instances, descriptive statistics are presented for both raw and categorical versions of features. For the longitudinal analyses, only those knees with complete and available baseline and 24-months data for the respective feature were included. Weighted kappa statistics were applied to determine inter- and intra-observer reliability for baseline and change over time. All analyses were conducted using SPSS 27 (IBM Corporation, Armonk, NY).

## Results

### Demographics

Of the 297 IMI-APPROACH participants, 289 had a readable baseline scan and at least one feature assessable (cartilage: *n* = 286, BML: *n* = 289, osteophytes: *n* = 285, meniscus: *n* = 278, inflammation: *n* = 287). There were 223 women (77.2%). Participants were on average 66.6 ± 7.1 years old and had a body mass index (BMI) of 28.1 ± 5.3 kg/m^2^. Mean knee injury and osteoarthritis outcome score (KOOS) symptom score was 69.5 ± 17.2, mean KOOS pain score was 66.4 ± 18.8 and mean KOOS function score was 69.1 ± 19.9. Mean numeric rating scale (NRS) pain score was 4.6 ± 2.7. A considerable proportion of the knees had no definite radiographic OA (44.6%, KL 0: *n* = 52; KL1: *n* = 77), but the majority (55.3%) of the knees had definite signs of radiographic OA (KL 2: *n* = 65 KL 3: *n* = 84, KL 4: *n* = 11). Medial joint space narrowing (JSN) was more frequent (47.8%) than lateral JSN (16.3%). Additional baseline characteristics of the cohort are presented in Table 1 and have been reported in detail previously [13].

**Table 1:** Demographic Characteristics of the Study Sample

		Mean/N	SD / %
Age	(years)	66.6	7.1
BMI	(kg/m^2^)	28.1	5.3
Side	Left	123	42.6
	Right	166	57.4
Sex	Female	223	77.2
	Male	66	22.8
Kellgren-Lawrence Grade	0	52	18.0
	1	77	26.6
	2	65	22.5
	3	84	29.1
	4	11	3.8
Medial joint space narrowing (3 missing)	0	151	52.2
	1	75	26.0
	2	40	13.8
	3	20	6.9
Lateral joint space narrowing (3 missing)	0	242	83.7
	1	24	8.3
	2	17	5.9
	3	3	1.0
Radiographic osteoarthritis	No	129	44.6
	Yes	160	55.4

*BMI* Body mass index, *N* Number, *SD* Standard deviation

### Reliability

Summarizing the intra- and inter-reader results for the baseline assessment, all of the measures showed at least substantial agreement ranging between 0.71 for maximum cartilage area extent on a knee level (intra-reader) and 1.00 for several features. Change was relatively rare and the reliability results of longitudinal data showed larger variation. Tables 2 and 3 give a detailed overview of the cross-sectional and longitudinal reliability results. Appendix 2 reports the frequencies of change for the reliability readings.

**Table 2:** Intra- and Inter-reader Reliability APPROACH MOAKS Assessment (Baseline)

*N* = 2		**Intra-observer**				**Inter-observer**			
		**Weighted Kappa**	**Std. Error**	**95% CI**		**Weighted Kappa**	**Std. Error**	**95% CI**	
**Maximum cartilage score**	**MFTJ**	0.79	0.08	0.62	0.95	0.89	0.05	0.79	0.98
	**LFTJ**	0.95	0.03	0.89	1.01	0.96	0.02	0.91	1.01
	**PFJ**	0.88	0.07	0.75	1.02	0.76	0.09	0.57	0.94
	**Knee**	0.75	0.09	0.58	0.92	0.85	0.06	0.73	0.97
**Maximum cartilage area score**	**MFTJ**	0.88	0.07	0.74	1.01	0.82	0.10	0.62	1.02
	**LFTJ**	1.00	0.00	1.00	1.00	0.96	0.04	0.89	1.04
	**PFJ**	0.87	0.09	0.70	1.04	0.72	0.15	0.43	1.02
	**Knee**	0.71	0.14	0.43	0.98	0.76	0.12	0.52	0.99
**Maximum cartilage full thickness score**	**MFTJ**	0.81	0.08	0.66	0.96	0.82	0.10	0.62	1.02
	**LFTJ**	0.90	0.07	0.77	1.03	0.90	0.06	0.78	1.02
	**PFJ**	1.00	0.00	1.00	1.00	0.88	0.08	0.72	1.04
	**Knee**	0.88	0.07	0.75	1.01	0.88	0.07	0.75	1.01
**Number of cartilage subregions with score > 0**	**MFTJ**	0.95	0.03	0.89	1.02	0.95	0.03	0.89	1.02
	**LFTJ**	1.00	0.00	1.00	1.00	0.97	0.03	0.91	1.03
	**PFJ**	0.84	0.08	0.69	0.99	0.92	0.06	0.81	1.03
	**Knee**	0.92	0.03	0.85	0.98	0.94	0.03	0.88	0.99
**Maximum BML size score**	**MFTJ**	0.94	0.06	0.82	1.06	1.00	0.00	1.00	1.00
	**LFTJ**	1.00	0.00	1.00	1.00	1.00	0.00	1.00	1.00
	**PFJ**	1.00	0.00	1.00	1.00	0.91	0.09	0.74	1.08
	**Knee**	0.95	0.05	0.86	1.04	1.00	0.00	1.00	1.00
**Number of regions with BML score > 0**	**MFTJ**	0.97	0.03	0.91	1.03	0.97	0.03	0.91	1.03
	**LFTJ**	1.00	0.00	1.00	1.00	0.89	0.06	0.77	1.01
	**PFJ**	1.00	0.00	1.00	1.00	0.86	0.10	0.67	1.04
	**Knee**	0.98	0.02	0.93	1.02	0.88	0.05	0.79	0.97
**Maximum osteophyte score**	**MFTJ**	0.95	0.05	0.86	1.04	0.77	0.10	0.57	0.96
	**LFTJ**	0.84	0.07	0.71	0.98	0.74	0.09	0.58	0.91
	**PFJ**	0.84	0.08	0.69	1.00	0.80	0.10	0.60	0.99
	**Knee**	0.91	0.06	0.79	1.03	0.91	0.06	0.79	1.03
**Meniscus morphology score (range 0–8)**	**MFTJ**	0.93	0.07	0.80	1.06	0.90	0.06	0.79	1.01
	**LFTJ**	0.93	0.05	0.83	1.03	0.83	0.06	0.71	0.95
**Meniscus morphology score (range 0:no/signal,1:tear,2:maceration)**	**MFTJ**	0.95	0.05	0.84	1.05	0.89	0.07	0.75	1.03
	**LFTJ**	1.00	0.00	1.00	1.00	0.94	0.06	0.83	1.05
**Meniscus extrusion (range 0–3)**	**MFTJ**	0.96	0.04	0.88	1.04	0.92	0.06	0.81	1.03
	**LFTJ**	0.88	0.08	0.72	1.03	0.94	0.07	0.81	1.07
**Meniscus extrusion (0/1 vs 2/3)**	**MFTJ**	1.00	0.00	1.00	1.00	0.90	0.10	0.71	1.09
	**LFTJ**	1.00	0.00	1.00	1.00	1.00	0.00	1.00	1.00
**Hoffa synovitis**	**Knee**	0.85	0.10	0.65	1.05	0.87	0.09	0.69	1.04
**Effusion synovitis**	**Knee**	1.00	0.00	1.00	1.00	0.89	0.08	0.74	1.04

*MFTJ* Medial tibio-femoral joint, *LFTJ* lateral tibio-femoral joint, *PFJ* Patellofemoral joint, *Std* Standard

**Table 3:** Intra- and Inter-reader Reliability APPROACH MOAKS Assessment (Change)

*N* = 20		**Intra-observer**				**Inter-observer**			
		**Weighted Kappa**	**Std. Error**	**95% CI**		**Weighted Kappa**	**Std. Error**	**95% CI**	
**Maximum increase in MOAKS cartilage score**	**MFTJ**	0.67	0.17	0.34	1.01	0.37	0.24	-0.10	0.84
	**LFTJ**	1.00	0.00	1.00	1.00	0.68	0.23	0.22	1.14
	**PFJ**	0.58	0.20	0.19	0.97	0.38	0.22	-0.05	0.81
	**Knee**	0.73	0.16	0.43	1.04	0.56	0.19	0.19	0.92
**Maximum increase in cartilage area score**	**MFTJ**	0.64	0.33	0.01	1.28	0.36	0.29	-0.20	0.93
	**LFTJ**	1.00	0.00	1.00	1.00	0.64	0.33	0.01	1.28
	**PFJ**	0.63	0.20	0.24	1.01	0.41	0.21	-0.01	0.83
	**Knee**	0.76	0.16	0.45	1.07	0.56	0.21	0.14	0.97
**Maximum increase in cartilage full thickness score**	**MFTJ**	0.76	0.16	0.45	1.07	0.47	0.22	0.04	0.90
	**LFTJ**	1.00	0.00	1.00	1.00	0.72	0.21	0.31	1.12
	**PFJ**	0.48	0.25	-0.02	0.98	0.32	0.30	-0.27	0.90
	**Knee**	0.74	0.15	0.46	1.03	0.65	0.16	0.33	0.97
**Maximum increase in BML size score**	**MFTJ**	0.76	0.17	0.43	1.10	0.54	0.15	0.23	0.84
	**LFTJ**	0.88	0.13	0.62	1.13	0.77	0.18	0.42	1.12
	**PFJ**	0.48	0.25	-0.02	0.98	0.23	0.26	-0.29	0.75
	**Knee**	0.87	0.10	0.68	1.06	0.79	0.10	0.60	0.99
**Change in number of regions with BML score > 0**	**MFTJ**	0.63	0.23	0.18	1.08	0.52	0.22	0.08	0.96
	**LFTJ**	0.65	0.27	0.12	1.18	0.53	0.17	0.20	0.87
	**PFJ**	0.79	0.21	0.38	1.19	0.47	0.32	-0.15	1.09
	**Knee**	0.55	0.22	0.13	0.98	0.57	0.17	0.24	0.89
**Maximum increase in osteophyte score**	**MFTJ**^**a**^	n/a	n/a	n/a	n/a	n/a	n/a	n/a	n/a
	**LFTJ**	0.64	0.33	0.01	1.28	1.00	0.00	1.00	1.00
	**PFJ**	0.00	0.00	0.00	0.00	0.00	0.00	0.00	0.00
	**Knee**	0.46	0.31	-0.14	1.06	0.61	0.25	0.11	1.10
**Max increase in meniscus morphology score (range 0…8)**	**MFTJ**	0.84	0.11	0.62	1.06	0.84	0.17	0.52	1.17
	**LFTJ**	0.63	0.29	0.07	1.19	0.24	0.20	-0.14	0.63
**Max increase in meniscus extrusion (range 0…3)**	**MFTJ**	1.00	0.00	1.00	1.00	0.00	0.00	0.00	0.00
	**LFTJ**	0.00	0.00	0.00	0.00	0.79	0.23	0.33	1.25
**Max increase in hoffa synovitis**	**Knee**	0.64	0.33	0.00	1.28	0.31	0.30	-0.27	0.90
**Max increase in effusion synovitis**	**Knee**	0.85	0.11	0.64	1.06	0.44	0.16	0.14	0.75

*MFTJ* medial tibio-femoral joint, *LFTJ* lateral tibio-femoral joint, *PFJ* Patellofemoral joint, *Std* standard

^a^In none of the knees an increase in osteophyte size in the medial tibio-femoral joint (MFTJ) was observed (both readers)

### Cartilage

Regarding baseline frequencies for cartilage, 4.9% of knees had a maximum baseline cartilage score (area extent) of 1, 52.1% of 2 and 40.6% of 3. Only 2.4% did not have any cartilage damage in any of the three compartments. In the ROA subgroup markedly more knees showed higher-grade cartilage damage compared to those knees without ROA (*p* = 0.0000). Regarding the full-thickness component of the MOAKS cartilage score, the respective numbers were 24.8% (grade 0), 16.4% (grade 1), 41.3% (grade 2) and 17.5% (grade 3). Details including number of subregions per knee and compartment affected by cartilage damage at baseline and differences between the ROA and no ROA subgroups are presented in Tables 4 and 5 and Appendix 3. Any change in total cartilage MOAKS score was seen in 53.1% of the entire sample considering only full-grade changes and in 73.9% including full-grade and within-grade changes. Any medial cartilage progression was seen in 23.9% and any lateral progression on 22.1% while any change in the PFJ was observed in 25.7%. Detailed results of change in cartilage for the number of subregions showing any increase (full-grade increase and full-grade plus within-grade increase) in total MOAKS score are presented in Table 6, and separately for the area-extent and full thickness dimensions in Appendix 4 and 5.

**Table 4:** Baseline cartilage damage (area extent score)

N = 286			All knees		No ROA		ROA		*P*-value
			Frequency	Percent	Frequency	Percent	Frequency	Percent	
Knee	Max score	0	7	2.4	7	5.4	0	0.0	0.0000
		1	14	4.9	14	10.9	0	0.0	
		2	149	52.1	93	72.1	56	35.7	
		3	116	40.6	15	11.6	101	64.3	
	Number of subregions	0	7	2.4	7	5.4	0	0.0	0.0000
		1	22	7.7	21	16.3	1	0.6	
		2	25	8.7	23	17.8	2	1.3	
		3	21	7.3	18	14.0	3	1.9	
		4	38	13.3	24	18.6	14	8.9	
		5	31	10.8	17	13.2	14	8.9	
		6	31	10.8	8	6.2	23	14.6	
		7	37	12.9	8	6.2	29	18.5	
		8	26	9.1	2	1.6	24	15.3	
		9	14	4.9	1	0.8	13	8.3	
		10	14	4.9	0	0.0	14	8.9	
		11 +	20	7.0	0	0.0	20	12.7	
MFTJ	Max score	0	67	23.4	56	43.4	11	7.0	0.0000
		1	14	4.9	8	6.2	6	3.8	
		2	146	51.0	61	47.3	85	54.1	
		3	59	20.6	4	3.1	55	35.0	
	Number of sub regions	0	67	23.4	56	43.4	11	7.0	0.0000
		1	55	19.2	34	26.4	21	13.4	
		2	57	19.9	29	22.5	28	17.8	
		3	44	15.4	8	6.2	36	22.9	
		4	30	10.5	0	0.0	30	19.1	
		5	33	11.5	2	1.6	31	19.7	
LFTJ	Max score	0	128	44.8	76	58.9	52	33.1	0.0000
		1	38	13.3	19	14.7	19	12.1	
		2	97	33.9	34	26.4	63	40.1	
		3	23	8.0	0	0.0	23	14.6	
	Number of subregions	0	128	44.8	76	58.9	52	33.1	0.0000
		1	68	23.8	37	28.7	31	19.7	
		2	35	12.2	11	8.5	24	15.3	
		3	16	5.6	5	3.9	11	7.0	
		4	20	7.0	0	0.0	20	12.7	
		5	19	6.6	0	0.0	19	12.1	
PFJ	Max score	0	27	9.4	19	14.7	8	5.1	0.0000
		1	35	12.2	23	17.8	12	7.6	
		2	163	57.0	74	57.4	89	56.7	
		3	61	21.3	13	10.1	48	30.6	
	Number of subregions	0	27	9.4	19	14.7	8	5.1	0.0000
		1	54	18.9	36	27.9	18	11.5	
		2	73	25.5	35	27.1	38	24.2	
		3	75	26.2	31	24.0	44	28.0	
		4	57	19.9	8	6.2	49	31.2

**Table 5:** Baseline cartilage damage (full thickness score)

*N* = 286			All knees		No ROA		ROA		*P*-value
			Frequency	Percent	Frequency	Percent	Frequency	Percent	
Knee	Max score	0	71	24.8	61	47.3	10	6.4	0.0000
		1	47	16.4	27	20.9	20	12.7	
		2	118	41.3	39	30.2	79	50.3	
		3	50	17.5	2	1.6	48	30.6	
	Number of regions	0	71	24.8	61	47.3	10	6.4	0.0000
		1	51	17.8	31	24.0	20	12.7	
		2	43	15.0	23	17.8	20	12.7	
		3	43	15.0	8	6.2	35	22.3	
		4	31	10.8	6	4.7	25	15.9	
		5	22	7.7	0	0.0	22	14.0	
		6 +	25	8.7	0	0.0	25	15.9	
MFTJ	Max score	0	186	65.0	114	88.4	72	45.9	0.0000
		1	34	11.9	9	7.0	25	15.9	
		2	49	17.1	6	4.7	43	27.4	
		3	17	5.9	0	0.0	17	10.8	
	Number of regions	0	186	65.0	114	88.4	72	45.9	0.0000
		1	50	17.5	13	10.1	37	23.6	
		2	17	5.9	1	0.8	16	10.2	
		3	14	4.9	1	0.8	13	8.3	
		4 +	19	6.6	0	0.0	19	12.1	
LFTJ	Max score	0	200	69.9	110	85.3	90	57.3	0.0000
		1	35	12.2	12	9.3	23	14.6	
		2	41	14.3	7	5.4	34	21.7	
		3	10	3.5	0	0.0	10	6.4	
	Number of regions	0	200	69.9	110	85.3	90	57.3	0.0000
		1	42	14.7	16	12.4	26	16.6	
		2	14	4.9	3	2.3	11	7.0	
		3	12	4.2	0	0.0	12	7.6	
		4 +	18	6.3	0	0.0	18	11.5	
PFJ	Max score	0	122	42.7	73	56.6	49	31.2	0.0000
		1	58	20.3	26	20.2	32	20.4	
		2	81	28.3	28	21.7	53	33.8	
		3	25	8.7	2	1.6	23	14.6	
	Number of regions	0	122	42.7	73	56.6	49	31.2	0.0000
		1	71	24.8	34	26.4	37	23.6	
		2	62	21.7	16	12.4	46	29.3	
		3	23	8.0	5	3.9	18	11.5	
		4	8	2.8	1	0.8	7	4.5

**Table 6:** Cartilage damage change – any MOAKS worsening (baseline to 24 months)

*N* = 226			All knees		No ROA		ROA		*P*-value
			Frequency	Percent	Frequency	Percent	Frequency	Percent	
Worsening—total MOAKS cartilage score (only full-grade worsening)									
Knee	None vs. any	0	106	46.9	69	63.3	37	31.6	0.0000
		≥ 1	120	53.1	43	36.7	80	68.4	
	Number of regions	1	60	26.5	26	23.9	34	29.1	
		2	35	15.5	9	8.3	26	22.2	
		3	17	7.5	3	2.8	14	12.0	
		4	6	2.7	2	1.8	4	3.4	
		5	2	0.9	0	0.0	2	1.7	
MFTJ	None vs. any	0	172	76.1	96	88.1	76	65.0	0.0000
		≥ 1	54	23.9	13	11.9	41	35.0	
	Number of regions	1	40	17.7	11	10.1	29	24.8	
		2	12	5.3	1	0.9	11	9.4	
		3	2	0.9	1	0.9	1	0.9	
LFTJ	None vs. any	0	176	77.9	99	90.8	77	65.8	0.0000
		≥ 1	50	22.1	10	9.2	40	34.2	
	Number of regions	1	35	15.5	8	7.3	27	23.1	
		2	11	4.9	2	1.8	9	7.7	
		3	3	1.3	0	0.0	3	2.6	
		4	1	0.4	0	0.0	1	0.9	
PFJ	None vs. any	0	168	74.3	83	76.1	85	72.6	0.5176
		≥ 1	58	25.7	26	23.9	32	27.4	
	Number of regions	1	43	19.0	20	18.3	23	19.7	
		2	13	5.8	5	4.6	8	6.8	
		3	2	0.9	1	0.9	1	0.9	
Worsening—total score including within-grade worsening									
Knee	None vs. any	0	85	37.6	61	56.0	24	20.5	0.0000
		≥ 1	141	73.9	48	44.0	93	79.5	
	Number of regions	1	59	26.1	30	27.5	29	24.8	
		2	43	19.0	12	11.0	31	26.5	
		3	29	12.8	4	3.7	25	21.4	
		4	7	3.1	2	1.8	5	4.3	
		5	3	1.3	0	0.0	3	2.6	
MFTJ	None vs. any	0	154	68.1	93	85.3	61	52.1	0.0000
		≥ 1	72	31.9	16	14.7	56	47.9	
	Number of regions	1	50	22.1	13	11.9	37	31.6	
		2	16	7.1	2	1.8	14	12.0	
		3	6	2.7	1	0.9	5	4.3	
LFTJ	None vs. any	0	168	74.3	97	89.0	71	60.7	0.0000
		≥ 1	58	25.7	12	11.0	46	39.3	
	Number of regions	1	38	16.8	10	9.2	28	23.9	
		2	16	7.1	2	1.8	14	12.0	
		3	3	1.3	0	0.0	3	2.6	
		4	1	0.4	0	0.0	1	0.9	
PFJ	None vs. any	0	154	68.1	77	70.6	77	65.8	0.3948
		≥ 1	72	31.9	32	29.4	40	34.2	
	Number of regions	1	54	23.9	25	22.9	29	24.8	
		2	16	7.1	6	5.5	10	8.5	
		3	2	0.9	1	0.9	1	0.9	

### BMLs

BMLs were observed in 77.5% of all knees at baseline, with 31.5% having a maximum score of 1, 27.0% a maximum score of 2 and 19% a maximum score of 3. BMLs were more commonly observed medially (35.3%) compared to the lateral compartment (23.5%), but were most prevalent in the patellofemoral joint (57.4%). The proportion of knees with any BMLs was markedly higher in the ROA (89.4%) compared to the no ROA subgroup (62.8%). The detailed results for baseline BMLs including the comparison between ROA and no ROA knees are shown in Table 7. Number of subregions showing BML change over time ranged from -4 to + 4 reflecting the fluctuation of BML, with the majority of knees having the same number of subregions affected by BMLs at baseline and follow up (60.3%). While for the medial and lateral compartments numbers of subregions with improvement and worsening were similar (9.9% and 9.5% medial, 7.3% and 5.6% lateral), for the PFJ more improvement was observed compared to worsening (15.5% vs. 9.0%), albeit not statistically significant. Including within-grade changes, the number of knees showing BML worsening increased from 42.2% to 55.6%. More details on BML change are presented in Table 8 and Appendix 6, 7 and 8.

**Table 7:** Baseline frequencies of bone marrow lesions

*N* = 289			All knees		No ROA		ROA		*P*-value
			Frequency	Percent	Frequency	Percent	Frequency	Percent	
Knee	Number of regions	0	65	22.5	48	37.2	17	10.6	0.0000
		1	39	13.5	26	20.2	13	8.1	
		2	60	20.8	28	21.7	32	20.0	
		3	55	19.0	18	14.0	37	23.1	
		4	37	12.8	7	5.4	30	18.8	
		5	18	6.2	2	1.6	16	10.0	
		6 +	15	5.2	0	0.0	15	9.4	
	Maximum score	0	65	22.5	48	37.2	17	10.6	0.0000
		1	91	31.5	44	34.1	47	29.4	
		2	78	27.0	24	18.6	54	33.8	
		3	55	19.0	13	10.1	42	26.3	
MFTJ	Number of regions	0	187	64.7	106	82.2	81	50.6	0.0000
		1	44	15.2	15	11.6	29	18.1	
		2	19	6.6	7	5.4	12	7.5	
		3	18	6.2	1	0.8	17	10.6	
		4 +	21	7.3	0	0.0	21	13.1	
	Maximum score	0	187	64.7	106	82.2	81	50.6	0.0000
		1	51	17.6	15	11.6	36	22.5	
		2	28	9.7	5	3.9	23	14.4	
		3	23	8.0	3	2.3	20	12.5	
LFTJ	Number of regions	0	221	76.5	114	88.4	107	66.9	0.0000
		1	30	10.4	12	9.3	18	11.3	
		2	10	3.5	3	2.3	7	4.4	
		3	13	4.5	0	0.0	13	8.1	
		4 +	15	5.2	0	0.0	15	9.4	
	Maximum score	0	221	76.5	114	88.4	107	66.9	0.0000
		1	32	11.1	13	10.1	19	11.9	
		2	21	7.3	2	1.6	19	11.9	
		3	15	5.2	0	0.0	15	9.4	
PFJ	Number of regions	0	123	42.6	60	46.5	63	39.4	0.2901
		1	66	22.8	27	20.9	39	24.4	
		2	71	24.6	30	23.3	41	25.6	
		3 +	29	10.0	12	9.3	17	10.6	
	Maximum score	0	123	42.6	60	46.5	63	39.4	0.2806
		1	88	30.4	37	28.7	51	31.9	
		2	57	19.7	22	17.1	35	21.9	
		3	21	7.3	10	7.8	11	6.9

**Table 8:** BML change overview baseline to 24 months follow-up

*N* = 232			All knees		No ROA		ROA		P
			Frequency	Percent	Frequency	Percent	Frequency	Percent	
Change in number of subregions having BMLs: #subregions with BMLs at follow-up—#subregions with BMLs at baseline									
Knee	Number of regions	-4	2	0.9	1	0.9	1	0.8	0.2606
		-2	10	4.3	3	2.7	7	5.7	
		-1	41	17.7	16	14.5	25	20.5	
		0	140	60.3	83	75.5	57	46.7	
		1	33	14.2	5	4.5	28	23.0	
		2	5	2.2	2	1.8	3	2.5	
		4	1	0.4	0	0.0	1	0.8	
MFTJ	Number of regions	-1	23	9.9	6	5.5	17	13.9	0.4520
		0	187	80.6	101	91.8	86	70.5	
		1	20	8.6	3	2.7	17	13.9	
		2	2	0.9	0	0.0	2	1.6	
LFTJ	Number of regions	-3	1	0.4	0	0.0	1	0.8	0.9946
		-2	1	0.4	0	0.0	1	0.8	
		-1	15	6.5	4	3.6	11	9.0	
		0	202	87.1	104	94.5	98	80.3	
		1	10	4.3	2	1.8	8	6.6	
		2	2	0.9	0	0.0	2	1.6	
		3	1	0.4	0	0.0	1	0.8	
PFJ	Number of regions	-3	1	0.4	1	0.9	0	0.0	0.8211
		-2	4	1.7	2	1.8	2	1.6	
		-1	31	13.4	11	10.0	20	16.4	
		0	175	75.4	90	81.8	85	69.7	
		1	20	8.6	6	5.5	14	11.5	
		2	1	0.4	0	0.0	1	0.8	
Change in number of regions (categories)									
Knee	Number of regions	Improvement	53	22.8	20	18.2	33	27.0	0.2484
		Stable	140	60.3	83	75.5	57	46.7	
		Worsening (1)	33	14.2	5	4.5	28	23.0	
		Worsening (2 +)	6	2.6	2	1.8	4	3.3	
MFTJ	Number of regions	Improvement	23	9.9	6	5.5	17	13.9	0.4520
		Stable	187	80.6	101	91.8	86	70.5	
		Worsening (1)	20	8.6	3	2.7	17	13.9	
		Worsening (2 +)	2	0.9	0	0.0	2	1.6	
LFTJ	Number of regions	Improvement	17	7.3	4	3.6	13	10.7	0.9839
		Stable	202	87.1	104	94.5	98	80.3	
		Worsening (1)	10	4.3	2	1.8	8	6.6	
		Worsening (2 +)	3	1.3	0	0.0	3	2.5	
PFJ	Number of regions	Improvement	36	15.5	14	12.7	22	18.0	0.8616
		Stable	175	75.4	90	81.8	85	69.7	
		Worsening (1)	20	8.6	6	5.5	14	11.5	
		Worsening (2 +)	1	0.4	0	0.0	1	0.8	

### Osteophytes

The large majority of knees exhibited osteophytes at baseline with 45.3% having a maximum grade of 1, 24.2% a maximum grade of 2 and 20.4% a maximum grade of 3, which is shown in more detail in Table 9. Osteophyte worsening was rare with 20% showing an increase in number of locations affected by any osteophyte. 19.1% of knees showed an increase in osteophyte size by one grade and 0.9% by two grades (Appendix 9).

**Table 9:** Baseline Frequencies of Osteophytes

*N* = 285			All knees		No ROA		ROA		P
			Frequency	Percent	Frequency	Percent	Frequency	Percent	
Knee	Number of locations	0	29	10.2	28	22.0	1	0.6	0.0000
		1	43	15.1	40	31.5	3	1.9	
		2	36	12.6	31	24.4	5	3.2	
		3	19	6.7	10	7.9	9	5.7	
		4	21	7.4	7	5.5	14	8.9	
		5	12	4.2	1	0.8	11	7.0	
		6	19	6.7	4	3.1	15	9.5	
		7	24	8.4	3	2.4	21	13.3	
		8	24	8.4	2	1.6	22	13.9	
		9	18	6.3	1	0.8	17	10.8	
		10	15	5.3	0	0.0	15	9.5	
		11	14	4.9	0	0.0	14	8.9	
		12	11	3.9	0	0.0	11	7.0	
	Max score	0	29	10.2	28	22.0	1	0.6	0.0000
		1	129	45.3	92	72.4	37	23.4	
		2	69	24.2	6	4.7	63	39.9	
		3	58	20.4	1	0.8	57	36.1	
MFTJ	Number of locations	0	59	20.7	54	42.5	5	3.2	0.0000
		1	71	24.9	53	41.7	18	11.4	
		2	60	21.1	17	13.4	43	27.2	
		3	95	33.3	3	2.4	92	58.2	
	Max score	0	59	20.7	54	42.5	5	3.2	0.0000
		1	132	46.3	71	55.9	61	38.6	
		2	67	23.5	1	0.8	66	41.8	
		3	27	9.5	1	0.8	26	16.5	
LFTJ	Number of locations	0	103	36.1	87	68.5	16	10.1	0.0000
		1	68	23.9	30	23.6	38	24.1	
		2	64	22.5	8	6.3	56	35.4	
		3	50	17.5	2	1.6	48	30.4	
	Max score	0	103	36.1	87	68.5	16	10.1	0.0000
		1	94	33.0	35	27.6	59	37.3	
		2	45	15.8	5	3.9	40	25.3	
		3	43	15.1	0	0.0	43	27.2	
PFJ	Number of locations	0	95	33.3	76	59.8	19	12.0	0.0000
		1	48	16.8	29	22.8	19	12.0	
		2	41	14.4	13	10.2	28	17.7	
		3	26	9.1	5	3.9	21	13.3	
		4	34	11.9	3	2.4	31	19.6	
		5	24	8.4	1	0.8	23	14.6	
		6	17	6.0	0	0.0	17	10.8	
	Max score	0	95	33.3	76	59.8	19	12.0	0.0000
		1	128	44.9	48	37.8	80	50.6	
		2	42	14.7	3	2.4	39	24.7	
		3	20	7.0	0	0.0	20	12.7	

### Meniscus

Regarding baseline meniscal pathology (Table 10), 51.1% of knees had any damage in the medial compartment, and 23.4% had damage in the lateral compartment. Meniscal tears were seen in 17.6% medially and 10.4% laterally, and any meniscal maceration was seen in 33.5% medially and 12.9% laterally. Root tears were rare (3.2% medially and 0.7% laterally). Meniscal extrusion grade 2 or 3 was detected in 33.8% medially and 9% laterally. Change in meniscal damage was rare with 3.2% showing change in one category (from normal to tear or tear to maceration) and 0.9% in two categories (normal to maceration). Any increase in extrusion was seen in 10.9% medially and 2.3% laterally (Appendix 10).

**Table 10:** Baseline Frequencies of Meniscus Pathology

*N* = 278			All knees		No ROA		ROA		
			Frequency	Percent	Frequency	Percent	Frequency	Percent	*p*-value
MFTJ	Maximum score	0	46	16.5	35	27.8	11	7.2	0.0000
		1	90	32.4	55	43.7	35	23.0	
		2	22	7.9	11	8.7	11	7.2	
		3	9	3.2	7	5.6	2	1.3	
		4	18	6.5	8	6.3	10	6.6	
		6	89	32.0	10	7.9	79	52.0	
		7	4	1.4	0	0.0	4	2.6	
	Meniscus morphology	No/Signal	136	48.9	90	71.4	46	30.3	0.0000
		Tear	49	17.6	26	20.6	23	15.1	
		Maceration	93	33.5	10	7.9	83	54.6	
	Root tear	No	269	96.8	126	100.0	143	94.1	0.0056
		Yes	9	3.2	0	0.0	9	5.9	
	Maximum extrusion	0	112	40.3	76	60.3	36	23.7	0.0000
		1	72	25.9	37	29.4	35	23.0	
		2	59	21.2	12	9.5	47	30.9	
		3	35	12.6	1	0.8	34	22.4	
LFTJ	Maximum score	0	168	60.4	88	69.8	80	52.6	0.0002
		1	45	16.2	19	15.1	26	17.1	
		2	18	6.5	11	8.7	7	4.6	
		3	4	1.4	4	3.2	0	0.0	
		4	7	2.5	3	2.4	4	2.6	
		6	33	11.9	1	0.8	32	21.1	
		7	3	1.1	0	0.0	3	2.0	
	Meniscus morphology	No/Signal	213	76.6	107	84.9	106	69.7	0.0004
		Tear	29	10.4	18	14.3	11	7.2	
		Maceration	36	12.9	1	0.8	35	23.0	
	Root tear	No	276	99.3	126	100.0	150	98.7	0.1971
		Yes	2	0.7	0	0.0	2	1.3	
	Maximum extrusion	0	238	85.6	117	92.9	121	79.6	0.0009
		1	15	5.4	7	5.6	8	5.3	
		2	12	4.3	2	1.6	10	6.6	
		3	13	4.7	0	0.0	13	8.6	

### Inflammation

Concerning inflammatory features of OA at baseline, 33.4% had no Hoffa-synovitis, 49.8% had grade 1 Hoffa-synovitis and 16.7% had grade 2 or 3 Hoffa-synovitis. Effusion-synovitis was seen in 30.3% (grade1), 11.5% (Grade 2) and 4.2% (grade 3) respectively, 54.0% did not have any effusion-synovitis at baseline. Regarding change in inflammation, 7% showed improvement and 7.8% worsening of Hoffa-synovitis, while for effusion-synovitis these numbers were 10% and 22%, respectively. Details of baseline and change characteristics of inflammatory features of OA are presented in Table 11.

**Table 11:** Hoffa- and effusion-synovitis – baseline and change over 24 months

		All knees		No ROA		ROA		P
		Frequency	Percent	Frequency	Percent	Frequency	Percent	
Baseline								
Hoffa synovitis	0	96	33.4	57	44.2	39	24.7	0.0001
	1	143	49.8	60	46.5	83	52.5	
	2+	48	16.7	12	9.3	36	22.8	
Effusion synovitis	0	155	54.0	101	78.3	54	34.2	0.0000
	1	87	30.3	23	17.8	64	40.5	
	2	33	11.5	1	0.8	32	20.3	
	3	12	4.2	4	3.1	8	5.1	
Change in maximum score by grade								
Hoffa synovitis	-1	16	7.0	6	5.5	10	8.3	0.7526
	0	196	85.2	98	89.1	98	81.7	
	1	17	7.4	5	4.5	12	10.0	
	2	1	0.4	1	0.9	0	0.0	
Effusion synovitis	-2	1	0.4	1	0.9	0	0.0	0.7947
	-1	22	9.6	5	4.5	17	14.2	
	0	156	67.8	83	75.5	73	60.8	
	1	48	20.9	19	17.3	29	24.2	
	2	2	0.9	1	0.9	1	0.8	
	3	1	0.4	1	0.9	0	0.0	
Change in maximum score by category								
Hoffa synovitis	Improvement	16	7.0	6	5.5	10	8.3	0.7380
	Stable	196	85.2	98	89.1	98	81.7	
	Worsening	18	7.8	6	5.5	12	10.0	
Effusion synovitis	Improvement	23	10.0	6	5.5	17	14.2	0.8159
	Stable	156	67.8	83	75.5	73	60.8	
	Worsening	51	22.2	21	19.1	30	25.0	

## Discussion

We presented baseline data and change over 24-months follow-up of SQ-assessed MRI features including cartilage, BMLs, osteophytes, meniscal pathology and inflammatory features of OA in the IMI-APPROACH study. We found a wide range of structural pathologies at baseline and substantial and varying change of features over the two-year follow-up period. Knees with established radiographic OA showed more baseline pathologies and more worsening of structural tissue damage over time compared to knees without radiographic OA.

The IMI-APPROACH cohort was specifically designed to include patients with knees likely to show structural or symptomatic progression over a two-year follow-up period. Participants were recruited primarily from existing cohorts and machine-learning models were applied to estimate risk of progression. This study is a descriptive overview of structural tissue pathology and longitudinal change. We did not attempt to show superiority of SQ MRI assessment over other methods. IMI-APPROACH employs a multitude of imaging methods including but not limited to radiographic parameters of knee OA severity, quantitative MRI parameters for cartilage including thickness and volume, SQ MRI scoring of cartilaginous and non-cartilaginous tissues, advanced radiographic parameters such as bone shape analyses and subchondral bone architecture and high-resolution CT characterizing OA related bone and trabecular adaptations [13]. Aim of IMI-APPROACH was to explore these different methods not in a comparative fashion but rather use the complementary information to help reach the overarching aim of being able to define different subtypes of OA, which hopefully will result in a more targeted personalized treatment approach in the future.

When comparing the IMI-APROACH data to the FNIH study, another cohort designed to analyze different biomarkers (including imaging) predicting structural or symptomatic progression, we found that in IMI-APPROACH fewer knees showed worsening in BMLs but a higher number of knees showed progression in the cartilage full-thickness dimension [28]. In APPROACH 42% of knees showed worsening of BMLs in any subregion (59% including within-grade changes) while this number was 73% for the cases and 66% in the control group in the FNIH study (also including within-grade changes). Regarding cartilage damage worsening in FNIH, 59% of subjects had at least one subregion with worsening in area extent dimension of MOAKS including within-grade changes (52% controls vs. 73% cases), while 42% of subjects (24% controls vs. 58% cases) had at least one area with worsening in thickness (considering full grade changes only). In IMI-APPROACH these numbers were 46% (29% no radiographic OA vs. 62% radiographic OA cases) for area extent (including within-grade changes) and similarly 46% (28% no radiographic OA vs. 72% radiographic OA cases) for full-thickness changes (full grade changes only). Regarding inflammatory features of OA, in FNIH 10% of subjects experienced worsening of Hoffa-synovitis with more cases experiencing worsening than controls (17% vs. 6%). In APPROACH this number was similar with 8% overall and 6% for the no radiographic OA subgroup and 10% for radiographic OA cases. In FNIH, the effusion-synovitis score worsened in 41% of cases compared to 18% of controls. In IMI-APPROACH, this number was 22% for the entire sample (19% for the no radiographic OA vs 26% for the radiographic OA subgroups) [28]. The other analyzed parameters (e.g. meniscus and osteophytes) showed little change in both cohorts. Of note, including within-grade assessment increased the number of knees showing change in cartilage and BML parameters. While clinical validity of within-grade assessment has been shown previously, recently it was shown that knees with within-grade changes have larger quantitative cartilage loss compared to those not showing any SQ cartilage change [29]. While both studies focus on progression in symptoms and / or structure, they differ in regard to patient selection. While FNIH used an a priori definition of progression based on pain and increase in joint space narrowing (67% of knees showing either increase in pain, increase in joint space narrowing or both) and applied a retrospective analysis of a prospectively acquired dataset, the IMI-APPROACH project worked with prediction models based on machine learning and existing cohorts. Furthermore, IMI-APROACH included a larger number of patients without radiographic OA (45%) while in FNIH only a small subset did not have radiographic OA (12.5%) [8].

Recently data from the MOST study focusing on KL grade 2 and 3 knees reported cross-sectionally on frequencies of cartilage damage with a focus on spectrum of disease and variability of cartilage damage ranging from no damage to severe widespread damage [30]. In that study, 665 knees were included from participants with comparable demographics to our study. 79% of all knees (68% of KL2 and 94% of KL3 knees) showed widespread full-thickness cartilage damage. In IMI-APPROACH widespread full-thickness damage in at least one of the MOAKS subregions (i.e. MOAKS 3.2 and 3.3) was seen in 33% of all knees and in 6% of knees without ROA and 55% of knees with ROA. The additional compartmental analyses in IMI-APPROACH were performed for the area extent and full-thickness dimensions separately. Any baseline full-thickness damage grade 2 or 3 was seen in 41% respectively 18% for the entire cohort.

The machine-learning-based predicted structural progression probability score, which was used for enrollment of participants in the IMI-APPROACH project, was not part of our analysis as the current study focused descriptively on the baseline frequencies and change over time [15]. Prediction of progression will be a focus of additional work. A recent report found no associations between predicted s-score and actual observed quantitative cartilage thickness loss [31].

Reliability analysis was performed on 20 knees for a spectrum of structural disease severity in cross-sectional and longitudinal fashion. Longitudinal reliability has rarely been described for SQ scoring [32]. While reported values for cross-sectional assessment were in the range of expectation for very experienced readers as in the current study, the longitudinal values are highly influenced by the prevalence of observed change. For this reason, these values have to be interpreted with caution and we have presented actual change frequencies in addition for better interpretability.

Definition of change using SQ approaches is challenging as there are multiple possible definitions including subregional or maximum-grade approaches. Few studies are available that have focused on longitudinal change of MRI parameters using SQ assessment including the FNIH cohort [28]. Runhaar and colleagues suggested definitions of change that are largely similar to our description, but did not incorporate the number of subregions approach [33]. When assessing change over time using SQ MRI approaches, scores are often presented as mean values or summed over a defined anatomical region (usually compartment or knee) [34, 35]. For several reasons, such approaches are sub-optimal as sums are challenging to compare. For example, a sum of 5 acquired over 5 distinct subregions of a given compartment may mean one lesion with a grade 5 (considered severe) while 4 subregions will not exhibit any lesion (grade 0); alternatively, it may reflect grade 1 lesions across 5 subregions. This is the reason why we focused on a number of subregion- and maximum grade-approach. More work is desirable on the prognostic implications of having widespread low-grade involvement vs. focal severe damage.

Part of the study design was reading of MRIs in chronological order not blinded to time point, which is an established approach that increases sensitivity to change compared to blinded reading [36]. Only reading un-blinded to time point allows for the application of within-grade changes, which increases sensitivity for the detection of minor changes [23]. Analytic approaches using SQ MRI data should include the number of subregions or locations affected by tissue pathology, with further possible stratification using cut-offs related to severity of a certain feature. In addition, an approach considering maximum change over a pre-defined unit, such as a knee compartment or the entire joint, adds to the understanding of the amount of change observed, which may be lost using a summative approach.

Our study has several limitations that need mentioning. We presented the SQ MRI data in purely descriptive fashion and did not analyze prediction regarding presence of baseline features or concurrent change and subsequent structural or clinical outcomes, which will be focus of future work. Secondly, due to the wide range of structural disease severity the IMI-APPROACH study is not easily translatable to other datasets. Thirdly, two of the centers used 1.5 T MRI systems while the others employed 3 T systems. There is no data available regarding a direct comparison of SQ scoring of knee OA using 1.5 T vs. 3 T systems. Most of the available literature focused on assessment in the context of knee trauma and did not find marked differences [37–39]. One study compared a 1.0 T extremity system with a 1.5 T standard system regarding SQ knee OA assessment and found very comparable results [40]. While we cannot rule out that the image quality on the 3 T systems may have been slightly superior, an omission of relevant joint pathology due to the lower field strength at 1.5 T seems highly unlikely. Finally, we focused on SQ MRI assessment only and did not analyze correlations or concurrent changes with other measures of progression such as radiography or quantitative MRI.

## Conclusions

In summary, a wide range of MRI-detected structural pathologies was present in the IMI-APPROACH cohort. More severe changes, especially for BMLs, cartilage and meniscal damage were detected primarily among the ROA group suggesting that once disease is structurally established it progresses more likely than pre-radiographic OA. The role of structural predictors of progression that are also potential therapeutic targets for cartilage-anabolic or anti-catabolic approaches, anti-inflammatory agents or compounds targeting subchondral bone changes should be the focus of further evaluation. In addition, the complexity of the different SQ scoring systems needs to be considered when engaging in analyses focusing on change over time.

## Supplementary Information

**Additional file 1.**

**Additional file 2.**

**Additional file 3.**

**Additional file 4.**

**Additional file 5.**

**Additional file 6.**

**Additional file 7.**

**Additional file 8.**

**Additional file 9.**

**Additional file 10.**

## Data Availability

The data that support the findings of this study are available from the APPROACH Steering Committee but restrictions apply to the availability of these data, which were used under license for the current study, and so are not publicly available. Data are however available from Frank W. Roemer, M.D. (corresponding author), upon reasonable request and with permission of the APPROACH Steering Committee.
