# Supplementary material for: Structural tissue damage and 24-month progression of semi-quantitative MRI biomarkers of knee osteoarthritis in the IMI-APPROACH cohort
Source: BMC Musculoskelet Disord. 2022 Nov 17;23:988. doi: 10.1186/s12891-022-05926-1 (PMC9670371; doi:10.1186/s12891-022-05926-1)
Supplement: Supplementary file 1 — Additional file 1. [file 12891_2022_5926_MOESM1_ESM.docx]

**Appendix 1.** MRI Pulse Sequence Protocol Parameters

| Sequence | COR IW 2D FS TSE | TRA IW 2D FS TSE | SAG IW 2D TSE FS | COR T1 2D SE | SAG T1W 3D GRE WE | |
| --- | --- | --- | --- | --- | --- | --- |
| Plane | Coronal | Transversal | Sagittal | Coronal | Sagittal |  |
| FS | FS | FS | FS | FS | WE |  |
| Matrix (phase) | 256 | 256 | 256 | 307 | 512 |  |
| Matrix (frequency) | 256 | 256 | 256 | 384 | 512 |  |
| No. of slices | 32 | 32 | 32 | 24 | 80 |  |
| FOV (mm) | 150 | 150 | 150 | 150 | 150 |  |
| Slice thickness/gap (mm/mm) | 3/0.3 | 3/0.3 | 3/0.3 | 3/0.3 | 1.5/0 |  |
| Flip angle (() | 180 | 180 | 180 | 180 | 12 |  |
| TE/TR (ms/ms) | 29/3700 | 30/3600 | 30/3600 | 12/360 | 7/17 |  |
| No. excitations averaged | 1 | 1 | 1 | 1 | 1 |  |
| ETL | 8 | 8 | 8 | 1 | 1 |  |

FS – fat saturation; No. – number; FOV – field of view; TE – echo time; TR – repetition time; ETL –echo train length; COR –coronal; IW –intermediate-weighted; 2D –two-dimensional; TSE – turbo spin echo; TRA –transversal; SAG –sagittal; GRE –gradient echo; WE water excitation
