# Supplementary material for: Structural tissue damage and 24-month progression of semi-quantitative MRI biomarkers of knee osteoarthritis in the IMI-APPROACH cohort
Source: BMC Musculoskelet Disord. 2022 Nov 17;23:988. doi: 10.1186/s12891-022-05926-1 (PMC9670371; doi:10.1186/s12891-022-05926-1)
Supplement: Supplementary file 2 — Additional file 2. [file 12891_2022_5926_MOESM2_ESM.docx]

**Appendix 2.** Reliability APPROACH MOAKS Assessment (Frequencies of change)

|  | **Delta** | **Frequency** | **Percent** |
| --- | --- | --- | --- |
| **Maximum increase in MOAKS cartilage score - MFTJ** | .0 | 14 | 70.0 |
|  | .1 | 5 | 25.0 |
|  | 1.1 | 1 | 5.0 |
| **Maximum increase in MOAKS cartilage score – LFTJ** | .0 | 16 | 80.0 |
|  | .1 | 1 | 5.0 |
|  | .2 | 1 | 5.0 |
|  | 1.1 | 2 | 10.0 |
| **Maximum increase in MOAKS cartilage score – PFJ** | .0 | 14 | 70.0 |
|  | 1.0 | 3 | 15.0 |
|  | 1.1 | 3 | 15.0 |
| **Maximum increase in MOAKS cartilage score – Knee** | .0 | 9 | 45.0 |
|  | .1 | 4 | 20.0 |
|  | .2 | 1 | 5.0 |
|  | 1.0 | 2 | 10.0 |
|  | 1.1 | 4 | 20.0 |
| **Maximum increase in cartilage area score – MFTJ** | 0 | 19 | 95.0 |
|  | 1 | 1 | 5.0 |
| **Maximum increase in cartilage area score – LFTJ** | 0 | 18 | 90.0 |
|  | 1 | 2 | 10.0 |
| **Maximum increase in cartilage area score – PFJ** | 0 | 14 | 70.0 |
|  | 1 | 6 | 30.0 |
| **Maximum increase in cartilage area score – Knee** | 0 | 14 | 70.0 |
|  | 1 | 6 | 30.0 |
| **Maximum increase in cartilage full thickness score - MFTJ** | 0 | 14 | 70.0 |
|  | 1 | 6 | 30.0 |
| **Maximum increase in cartilage full thickness score - LFTJ** | 0 | 16 | 80.0 |
|  | 1 | 3 | 15.0 |
|  | 2 | 1 | 5.0 |
| **Maximum increase in cartilage full thickness score - PFJ** | 0 | 17 | 85.0 |
|  | 1 | 3 | 15.0 |
| **Maximum increase in cartilage full thickness score - Knee** | 0 | 11 | 55.0 |
|  | 1 | 8 | 40.0 |
|  | 2 | 1 | 5.0 |
| **Maximum increase in BML size score – MFTJ** | 0 | 17 | 85.0 |
|  | 1 | 2 | 10.0 |
|  | 3 | 1 | 5.0 |
| **Maximum increase in BML size score – LFTJ** | 0 | 17 | 85.0 |
|  | 1 | 1 | 5.0 |
|  | 2 | 2 | 10.0 |
| **Maximum increase in BML size score – PFJ** | 0 | 16 | 80.0 |
|  | 1 | 4 | 20.0 |
| **Maximum increase in BML size score – Knee** | 0 | 11 | 55.0 |
|  | 1 | 7 | 35.0 |
|  | 2 | 1 | 5.0 |

**Appendix 2.** Reliability APPROACH MOAKS Assessment (Frequencies of change) **- continued**

|  | **Delta** | **Frequency** | **Percent** |
| --- | --- | --- | --- |
| **Change in number of regions with BML score >0 – MFTJ** | -1 | 1 | 5.0 |
|  | 0 | 17 | 85.0 |
|  | 1 | 2 | 10.0 |
| **Change in number of regions with BML score >0 – LFTJ** | -1 | 1 | 5.0 |
|  | 0 | 17 | 85.0 |
|  | 1 | 1 | 5.0 |
|  | 3 | 1 | 5.0 |
| **Change in number of regions with BML score >0 – PFJ** | -1 | 1 | 5.0 |
|  | 0 | 18 | 90.0 |
|  | 1 | 1 | 5.0 |
| **Change in number of regions with BML score >0 – Knee** | -1 | 2 | 10.0 |
|  | 0 | 15 | 75.0 |
|  | 1 | 2 | 10.0 |
|  | 4 | 1 | 5.0 |
| **Maximum increase in osteophyte score – MFTJ** | 0 | 20 | 100.0 |
| **Maximum increase in osteophyte score – LFTJ** | 0 | 18 | 90.0 |
|  | 1 | 2 | 10.0 |
| **Maximum increase in osteophyte score – PFJ** | 0 | 19 | 95.0 |
|  | 1 | 1 | 5.0 |
| **Maximum increase in osteophyte score – Knee** | 0 | 17 | 85.0 |
|  | 1 | 3 | 15.0 |
| **Max increase in meniscus morphology score (range 0...8) – MFTJ** | 0 | 17 | 85.0 |
|  | 2 | 2 | 10.0 |
|  | 4 | 1 | 5.0 |
| **Max increase in meniscus morphology score (range 0...8) – LFTJ** | 0 | 17 | 85.0 |
|  | 2 | 2 | 10.0 |
|  | 6 | 1 | 5.0 |
| **Max increase in meniscus extrusion (range 0...3) – MFTJ** | 0 | 19 | 95.0 |
|  | 1 | 1 | 5.0 |
| **Max increase in meniscus extrusion (range 0...3) – LFTJ** | 0 | 18 | 90.0 |
|  | 1 | 1 | 5.0 |
|  | 2 | 1 | 5.0 |
| **Max increase in hoffa synovitis** | 0 | 17 | 85.0 |
|  | 1 | 2 | 10.0 |
| **Max increase in effusion synovitis** | -1 | 2 | 10.0 |
|  | 0 | 10 | 50.0 |
|  | 1 | 8 | 40.0 |
