# Supplementary material for: Structural tissue damage and 24-month progression of semi-quantitative MRI biomarkers of knee osteoarthritis in the IMI-APPROACH cohort
Source: BMC Musculoskelet Disord. 2022 Nov 17;23:988. doi: 10.1186/s12891-022-05926-1 (PMC9670371; doi:10.1186/s12891-022-05926-1)
Supplement: Supplementary file 3 — Additional file 3. [file 12891_2022_5926_MOESM3_ESM.docx]

**Appendix 3.** Baseline cartilage damage per knee (total MOAKS Score)

| MRI Feature N=286 | | | All knees | | No ROA | | ROA | | P-value |
| --- | --- | --- | --- | --- | --- | --- | --- | --- | --- |
|  |  |  | Frequency | Percent | Frequency | Percent | Frequency | Percent |  |
|  | Maximum MOAKS Score | 0.0 | 7 | 2.4 | 7 | 5.4 | 0 | 0.0 | 0.000 |
|  |  | 1.0 | 12 | 4.2 | 12 | 9.3 | 0 | 0.0 |  |
|  |  | 1.1 | 2 | 0.7 | 2 | 1.6 | 0 | 0.0 |  |
|  |  | 2.0 | 58 | 20.3 | 45 | 34.9 | 13 | 8.3 |  |
|  |  | 2.1 | 29 | 10.1 | 18 | 14.0 | 11 | 7.0 |  |
|  |  | 2.2 | 62 | 21.7 | 30 | 23.3 | 32 | 20.4 |  |
|  |  | 3.0 | 18 | 6.3 | 5 | 3.9 | 13 | 8.3 |  |
|  |  | 3.1 | 4 | 1.4 | 2 | 1.6 | 2 | 1.3 |  |
|  |  | 3.2 | 44 | 15.4 | 6 | 4.7 | 38 | 24.2 |  |
|  |  | 3.3 | 50 | 17.5 | 2 | 1.6 | 48 | 30.6 |  |
|  | Number of Regions involved | 0 | 7 | 2.4 | 7 | 5.4 | 0 | 0.0 | 0.000 |
|  |  | 1 | 22 | 7.7 | 21 | 16.3 | 1 | 0.6 |  |
|  |  | 2 | 25 | 8.7 | 23 | 17.8 | 2 | 1.3 |  |
|  |  | 3 | 21 | 7.3 | 18 | 14.0 | 3 | 1.9 |  |
|  |  | 4 | 38 | 13.3 | 24 | 18.6 | 14 | 8.9 |  |
|  |  | 5 | 31 | 10.8 | 17 | 13.2 | 14 | 8.9 |  |
|  |  | 6 | 31 | 10.8 | 8 | 6.2 | 23 | 14.6 |  |
|  |  | 7 | 37 | 12.9 | 8 | 6.2 | 29 | 18.5 |  |
|  |  | 8 | 26 | 9.1 | 2 | 1.6 | 24 | 15.3 |  |
|  |  | 9 | 14 | 4.9 | 1 | 0.8 | 13 | 8.3 |  |
|  |  | 10 | 14 | 4.9 | 0 | 0.0 | 14 | 8.9 |  |
|  |  | 11+ | 20 | 7.0 | 0 | 0.0 | 20 | 12.7 |  |
