# Supplementary material for: Structural tissue damage and 24-month progression of semi-quantitative MRI biomarkers of knee osteoarthritis in the IMI-APPROACH cohort
Source: BMC Musculoskelet Disord. 2022 Nov 17;23:988. doi: 10.1186/s12891-022-05926-1 (PMC9670371; doi:10.1186/s12891-022-05926-1)
Supplement: Supplementary file 4 — Additional file 4. [file 12891_2022_5926_MOESM4_ESM.docx]

**Appendix 4.** Cartilage damage change – MOAKS Area Extent-Component Worsening (baseline to 24 months)

| Worsening - area score **without** within-grade change; N=226 | | | | | | | | | |
| --- | --- | --- | --- | --- | --- | --- | --- | --- | --- |
|  |  |  | All knees | | No ROA | | ROA | | P-value |
|  |  |  | Frequency | Percent | Frequency | Percent | Frequency | Percent |  |
| Knee | None vs. any | 0 | 156 | 69.0 | 87 | 79.8 | 69 | 59.0 | 0.0010 |
|  |  | ≥1 | 70 | 31.0 | 22 | 20.2 | 48 | 70.9 |  |
|  | Number of regions | 1 | 49 | 21.7 | 15 | 13.8 | 34 | 29.1 |  |
|  |  | 2 | 16 | 7.1 | 5 | 4.6 | 11 | 9.4 |  |
|  |  | 3 | 3 | 1.3 | 2 | 1.8 | 1 | 0.9 |  |
|  |  | 4 | 1 | 0.4 | 0 | 0.0 | 1 | 0.9 |  |
|  |  | 5 | 1 | 0.4 | 0 | 0.0 | 1 | 0.9 |  |
| MFTJ | None vs. any | 0 | 202 | 89.4 | 102 | 93.6 | 100 | 85.5 | 0.0483 |
|  |  | ≥1 | 24 | 10.6 | 7 | 6.4 | 17 | 14.5 |  |
|  | Number of regions | 1 | 20 | 8.8 | 6 | 5.5 | 14 | 12.0 |  |
|  |  | 2 | 2 | 0.9 | 0 | 0.0 | 2 | 1.7 |  |
|  |  | 3 | 2 | 0.9 | 1 | 0.9 | 1 | 0.9 |  |
| LFTJ | None vs. any | 0 | 196 | 86.7 | 102 | 93.6 | 94 | 80.3 | 0.0034 |
|  |  | ≥1 | 30 | 13.3 | 7 | 6.4 | 23 | 19.7 |  |
|  | Number of regions | 1 | 25 | 11.1 | 6 | 5.5 | 19 | 16.2 |  |
|  |  | 2 | 5 | 2.2 | 1 | 0.9 | 4 | 3.4 |  |
| PFJ | None vs. any | 0 | 196 | 86.7 | 96 | 88.1 | 100 | 85.5 | 0.5402 |
|  |  | ≥1 | 30 | 13.3 | 13 | 11.9 | 17 | 14.5 |  |
|  | Number of regions | 1 | 26 | 11.5 | 12 | 11.0 | 14 | 12.0 |  |
|  |  | 2 | 4 | 1.8 | 1 | 0.9 | 3 | 2.6 |  |
| Worsening - area score **with** within-grade change | | | | | | | | | |
| Knee | None vs. any | 0 | 122 | 54.0 | 77 | 70.6 | 45 | 38.5 | 0.0000 |
|  |  | ≥1 | 104 | 46.0 | 32 | 29.4 | 72 | 61.5 |  |
|  | Number of regions | 1 | 54 | 23.9 | 21 | 19.3 | 33 | 28.2 |  |
|  |  | 2 | 41 | 18.1 | 9 | 8.3 | 32 | 27.4 |  |
|  |  | 3 | 6 | 2.7 | 2 | 1.8 | 4 | 3.4 |  |
|  |  | 4 | 2 | 0.9 | 0 | 0.0 | 2 | 1.7 |  |
|  |  | 5 | 1 | 0.4 | 0 | 0.0 | 1 | 0.9 |  |
| MFTJ | None vs. any | 0 | 179 | 79.2 | 99 | 90.8 | 80 | 68.4 | 0.0000 |
|  |  | ≥1 | 47 | 20.8 | 10 | 9.2 | 37 | 31.6 |  |
|  | Number of regions | 1 | 35 | 15.5 | 8 | 7.3 | 27 | 23.1 |  |
|  |  | 2 | 9 | 4.0 | 1 | 0.9 | 8 | 6.8 |  |
|  |  | 3 | 3 | 1.3 | 1 | 0.9 | 2 | 1.7 |  |
| LFTJ | None vs. any | 0 | 181 | 80.1 | 99 | 90.8 | 82 | 70.1 | 0.0001 |
|  |  | ≥1 | 45 | 19.9 | 10 | 9.2 | 35 | 29.9 |  |
|  | Number of regions | 1 | 37 | 16.4 | 9 | 8.3 | 28 | 23.9 |  |
|  |  | 2 | 8 | 3.5 | 1 | 0.9 | 7 | 6.0 |  |
| PFJ | None vs. any | 0 | 179 | 79.2 | 90 | 82.6 | 89 | 76.1 | 0.2306 |
|  |  | ≥1 | 47 | 20.8 | 19 | 17.4 | 28 | 23.9 |  |
|  | Number of regions | 1 | 42 | 18.6 | 17 | 15.6 | 25 | 21.4 |  |
|  |  | 2 | 5 | 2.2 | 2 | 1.8 | 3 | 2.6 |  |
