# Supplementary material for: Structural tissue damage and 24-month progression of semi-quantitative MRI biomarkers of knee osteoarthritis in the IMI-APPROACH cohort
Source: BMC Musculoskelet Disord. 2022 Nov 17;23:988. doi: 10.1186/s12891-022-05926-1 (PMC9670371; doi:10.1186/s12891-022-05926-1)
Supplement: Supplementary file 5 — Additional file 5. [file 12891_2022_5926_MOESM5_ESM.docx]

**Appendix 5.** Cartilage damage change – MOAKS Full-Thickness-Component Worsening (baseline to 24 months)

| Worsening - full thickness score **without** within-grade change, N=226 | | | | | | | | | |
| --- | --- | --- | --- | --- | --- | --- | --- | --- | --- |
|  |  |  | All knees | | No ROA | | ROA | | P-value |
|  |  |  | Frequency | Percent | Frequency | Percent | Frequency | Percent |  |
| Knee | None vs. any | 0 | 123 | 54.4 | 79 | 72.5 | 44 | 37.6 | 0.0000 |
|  |  | ≥1 | 103 | 45.5 | 30 | 27.5 | 73 | 62.4 |  |
|  | Number of regions | 1 | 67 | 29.6 | 22 | 20.2 | 45 | 38.5 |  |
|  |  | 2 | 27 | 11.9 | 6 | 5.5 | 21 | 17.9 |  |
|  |  | 3 | 6 | 2.7 | 1 | 0.9 | 5 | 4.3 |  |
|  |  | 4 | 2 | 0.9 | 1 | 0.9 | 1 | 0.9 |  |
|  |  | 5 | 1 | 0.4 | 0 | 0.0 | 1 | 0.9 |  |
| MFTJ | None vs. any | 0 | 186 | 82.3 | 100 | 91.7 | 86 | 73.5 | 0.0003 |
|  |  | ≥1 | 40 | 17.7 | 9 | 8.3 | 31 | 26.5 |  |
|  | Number of regions | 1 | 30 | 13.3 | 7 | 6.4 | 23 | 19.7 |  |
|  |  | 2 | 10 | 4.4 | 2 | 1.8 | 8 | 6.8 |  |
| LFTJ | None vs. any | 0 | 187 | 82.7 | 104 | 95.4 | 83 | 70.9 | 0.0000 |
|  |  | ≥1 | 39 | 17.3 | 5 | 4.6 | 34 | 29.1 |  |
|  | Number of regions | 1 | 32 | 14.2 | 4 | 3.7 | 28 | 23.9 |  |
|  |  | 2 | 5 | 2.2 | 1 | 0.9 | 4 | 3.4 |  |
|  |  | 3 | 1 | 0.4 | 0 | 0.0 | 1 | 0.9 |  |
|  |  | 4 | 1 | 0.4 | 0 | 0.0 | 1 | 0.9 |  |
| PFJ | None vs. any | 0 | 181 | 80.1 | 89 | 81.7 | 92 | 78.6 | 0.5925 |
|  |  | ≥1 | 45 | 19.9 | 20 | 18.3 | 25 | 21.4 |  |
|  | Number of regions | 1 | 37 | 16.4 | 16 | 14.7 | 21 | 17.9 |  |
|  |  | 2 | 8 | 3.5 | 4 | 3.7 | 4 | 3.4 |  |
| Worsening - full thickness score **with** within-grade change | | | | | | | | | |
| Knee | None vs. any | 0 | 111 | 49.1 | 78 | 71.6 | 33 | 28.2 | 0.0000 |
|  |  | ≥1 | 115 | 50.9 | 31 | 28.4 | 84 | 71.8 |  |
|  | Number of regions | 1 | 69 | 30.5 | 21 | 19.3 | 48 | 41.0 |  |
|  |  | 2 | 32 | 14.2 | 8 | 7.3 | 24 | 20.5 |  |
|  |  | 3 | 11 | 4.9 | 1 | 0.9 | 10 | 8.5 |  |
|  |  | 4 | 2 | 0.9 | 1 | 0.9 | 1 | 0.9 |  |
|  |  | 5 | 1 | 0.4 | 0 | 0.0 | 1 | 0.9 |  |
| MFTJ | None vs. any | 0 | 177 | 78.3 | 100 | 91.7 | 77 | 65.8 | 0.0000 |
|  |  | ≥1 | 49 | 21.7 | 9 | 8.3 | 40 | 34.2 |  |
|  | Number of regions | 1 | 35 | 15.5 | 7 | 6.4 | 28 | 23.9 |  |
|  |  | 2 | 14 | 6.2 | 2 | 1.8 | 12 | 10.3 |  |
| LFTJ | None vs. any | 0 | 183 | 81.0 | 104 | 95.4 | 79 | 67.5 | 0.0000 |
|  |  | ≥1 | 43 | 19 | 5 | 4.6 | 38 | 32.5 |  |
|  | Number of regions | 1 | 33 | 14.6 | 4 | 3.7 | 29 | 24.8 |  |
|  |  | 2 | 8 | 3.5 | 1 | 0.9 | 7 | 6.0 |  |
|  |  | 3 | 1 | 0.4 | 0 | 0.0 | 1 | 0.9 |  |
|  |  | 4 | 1 | 0.4 | 0 | 0.0 | 1 | 0.9 |  |
| PFJ | None vs. any | 0 | 175 | 77.4 | 87 | 79.8 | 88 | 75.2 | 0.4583 |
|  |  | ≥1 | 51 | 22.6 | 22 | 20.2 | 29 | 24.8 |  |
|  | Number of regions | 1 | 42 | 18.6 | 17 | 15.6 | 25 | 21.4 |  |
|  |  | 2 | 9 | 4.0 | 5 | 4.6 | 4 | 3.4 |  |
