# Supplementary material for: Structural tissue damage and 24-month progression of semi-quantitative MRI biomarkers of knee osteoarthritis in the IMI-APPROACH cohort
Source: BMC Musculoskelet Disord. 2022 Nov 17;23:988. doi: 10.1186/s12891-022-05926-1 (PMC9670371; doi:10.1186/s12891-022-05926-1)
Supplement: Supplementary file 6 — Additional file 6. [file 12891_2022_5926_MOESM6_ESM.docx]

**Appendix 6.** Maximum increase in BML Score from baseline to 24 months follow-up

| Maximum increase in BML score (Delta), Full grade only; N=232 | | | | | | | | | | | | | | | | |  |
| --- | --- | --- | --- | --- | --- | --- | --- | --- | --- | --- | --- | --- | --- | --- | --- | --- | --- |
| N=226 | | | All knees | | | | No ROA | | | | ROA | | | | | P-value | |
|  | | | Frequency | | Percent | | Frequency | | Percent | | Frequency | | Percent | | |  | |
| Knee | Maximum score δ | 0 | | 134 | | 57.8 | | 86 | | 78.2 | | 48 | | 39.3 | 0.0000 | |  |
|  |  | ≥1 | | 98 | | 42.2 | | 24 | | 21.8 | | 74 | | 60.7 |  |  |  |
|  |  | 1 | | 71 | | 30.6 | | 18 | | 16.4 | | 53 | | 43.4 |  |  |  |
|  |  | 2 | | 21 | | 9.1 | | 4 | | 3.6 | | 17 | | 13.9 |  |  |  |
|  |  | 3 | | 6 | | 2.6 | | 2 | | 1.8 | | 4 | | 3.3 |  |  |  |
| MFTJ | Maximum score δ | 0 | | 190 | | 81.9 | | 103 | | 93.6 | | 87 | | 71.3 | 0.0000 | |  |
|  |  | ≥1 | | 42 | | 18.1 | | 7 | | 6.4 | | 35 | | 28.7 |  |  |  |
|  |  | 1 | | 27 | | 11.6 | | 5 | | 4.5 | | 22 | | 18.0 |  |  |  |
|  |  | 2 | | 10 | | 4.3 | | 0 | | 0.0 | | 10 | | 8.2 |  |  |  |
|  |  | 3 | | 5 | | 2.2 | | 2 | | 1.8 | | 3 | | 2.5 |  |  |  |
| LFTJ | Maximum score δ | 0 | | 204 | | 87.9 | | 107 | | 97.3 | | 97 | | 79.5 | 0.0000 | |  |
|  |  | ≥1 | | 28 | | 12.1 | | 3 | | 2.7 | | 25 | | 20.5 |  |  |  |
|  |  | 1 | | 22 | | 9.5 | | 2 | | 1.8 | | 20 | | 16.4 |  |  |  |
|  |  | 2 | | 5 | | 2.2 | | 1 | | 0.9 | | 4 | | 3.3 |  |  |  |
|  |  | 3 | | 1 | | 0.4 | | 0 | | 0.0 | | 1 | | 0.8 |  |  |  |
| PFJ | Maximum score δ | 0 | | 179 | | 77.2 | | 93 | | 84.5 | | 86 | | 70.5 | 0.0133 | |  |
|  |  | ≥1 | | 53 | | 22.8 | | 17 | | 15.5 | | 36 | | 29.5 |  |  |  |
|  |  | 1 | | 46 | | 19.8 | | 14 | | 12.7 | | 32 | | 26.2 |  |  |  |
|  |  | 2 | | 7 | | 3.0 | | 3 | | 2.7 | | 4 | | 3.3 |  |  |  |
