# Supplementary material for: Structural tissue damage and 24-month progression of semi-quantitative MRI biomarkers of knee osteoarthritis in the IMI-APPROACH cohort
Source: BMC Musculoskelet Disord. 2022 Nov 17;23:988. doi: 10.1186/s12891-022-05926-1 (PMC9670371; doi:10.1186/s12891-022-05926-1)
Supplement: Supplementary file 7 — Additional file 7. [file 12891_2022_5926_MOESM7_ESM.docx]

**Appendix 7.** Worsening in BMLs from baseline to 24 months follow-up

| Number of regions with worsening (**without** within-grade changes, improvement=no change); N=232 | | | | | | | | | |
| --- | --- | --- | --- | --- | --- | --- | --- | --- | --- |
|  |  |  | All knees | | No ROA | | ROA | | P-value |
|  |  |  | Frequency | Percent | Frequency | Percent | Frequency | Percent |  |
| Knee | Number of regions | 0 | 134 | 57.8 | 86 | 78.2 | 48 | 39.3 | 0.0000 |
|  |  | ≥1 | 98 | 42.2 | 24 | 21.8 | 74 | 60.7 |  |
|  |  | 1 | 55 | 23.7 | 19 | 17.3 | 36 | 29.5 |  |
|  |  | 2 | 25 | 10.8 | 4 | 3.6 | 21 | 17.2 |  |
|  |  | 3 | 13 | 5.6 | 1 | 0.9 | 12 | 9.8 |  |
|  |  | 4 | 3 | 1.3 | 0 | 0.0 | 3 | 2.5 |  |
|  |  | 7 | 2 | 0.9 | 0 | 0.0 | 2 | 1.6 |  |
| MFTJ | Number of regions | 0 | 190 | 81.9 | 103 | 93.6 | 87 | 71.3 | 0.0000 |
|  |  | ≥1 | 42 | 18.1 | 7 | 6.4 | 35 | 28.7 |  |
|  |  | 1 | 26 | 11.2 | 6 | 5.5 | 20 | 16.4 |  |
|  |  | 2 | 12 | 5.2 | 1 | 0.9 | 11 | 9.0 |  |
|  |  | 3 | 2 | 0.9 | 0 | 0.0 | 2 | 1.6 |  |
|  |  | 4 | 1 | 0.4 | 0 | 0.0 | 1 | 0.8 |  |
|  |  | 5 | 1 | 0.4 | 0 | 0.0 | 1 | 0.8 |  |
| LFTJ | Number of regions | 0 | 204 | 87.9 | 107 | 97.3 | 97 | 79.5 | 0.0000 |
|  |  | ≥1 | 28 | 12.1 | 3 | 2.7 | 25 | 20.5 |  |
|  |  | 1 | 15 | 6.5 | 2 | 1.8 | 13 | 10.7 |  |
|  |  | 2 | 11 | 4.7 | 1 | 0.9 | 10 | 8.2 |  |
|  |  | 3 | 1 | 0.4 | 0 | 0.0 | 1 | 0.8 |  |
|  |  | 4 | 1 | 0.4 | 0 | 0.0 | 1 | 0.8 |  |
| PFJ | Number of regions | 0 | 179 | 77.2 | 93 | 84.5 | 86 | 70.5 | 0.0083 |
|  |  | ≥1 | 53 | 22.8 | 17 | 15.5 | 26 | 29.5 |  |
|  |  | 1 | 45 | 19.4 | 16 | 14.5 | 29 | 23.8 |  |
|  |  | 2 | 8 | 3.4 | 1 | 0.9 | 7 | 5.7 |  |
| Number of regions with worsening (**including** within-grade changes, improvement=no change): | | | | | | | | | |
| Knee | Number of regions | 0 | 103 | 44.4 | 73 | 66.4 | 30 | 24.6 | 0.0000 |
|  |  | ≥1 | 129 | 55.6 | 37 | 33.6 | 92 | 75.4 |  |
|  |  | 1 | 69 | 29.7 | 29 | 26.4 | 40 | 32.8 |  |
|  |  | 2 | 34 | 14.7 | 7 | 6.4 | 27 | 22.1 |  |
|  |  | 3 | 15 | 6.5 | 1 | 0.9 | 14 | 11.5 |  |
|  |  | 4 | 6 | 2.6 | 0 | 0.0 | 6 | 4.9 |  |
|  |  | 5 | 2 | 0.9 | 0 | 0.0 | 2 | 1.6 |  |
|  |  | 6 | 1 | 0.4 | 0 | 0.0 | 1 | 0.8 |  |
|  |  | 7 | 1 | 0.4 | 0 | 0.0 | 1 | 0.8 |  |
|  |  | 8 | 1 | 0.4 | 0 | 0.0 | 1 | 0.8 |  |
| MFTJ | Number of regions | 0 | 178 | 76.7 | 101 | 91.8 | 77 | 63.1 | 0.0000 |
|  |  | ≥1 | 54 | 23.3 | 9 | 8.2 | 45 | 36.9 |  |
|  |  | 1 | 35 | 15.1 | 8 | 7.3 | 27 | 22.1 |  |
|  |  | 2 | 13 | 5.6 | 1 | 0.9 | 12 | 9.8 |  |
|  |  | 3 | 3 | 1.3 | 0 | 0.0 | 3 | 2.5 |  |
|  |  | 4 | 2 | 0.9 | 0 | 0.0 | 2 | 1.6 |  |
|  |  | 5 | 1 | 0.4 | 0 | 0.0 | 1 | 0.8 |  |
| LFTJ | Number of regions | 0 | 191 | 82.3 | 104 | 94.5 | 87 | 71.3 | 0.0000 |
|  |  | ≥1 | 41 | 17.7 | 6 | 5.5 | 35 | 28.7 |  |
|  |  | 1 | 25 | 10.8 | 5 | 4.5 | 20 | 16.4 |  |
|  |  | 2 | 12 | 5.2 | 1 | 0.9 | 11 | 9.0 |  |
|  |  | 3 | 3 | 1.3 | 0 | 0.0 | 3 | 2.5 |  |
|  |  | 4 | 1 | 0.4 | 0 | 0.0 | 1 | 0.8 |  |
| PFJ | Number of regions | 0 | 156 | 67.2 | 84 | 76.4 | 72 | 59.0 | 0.0028 |
|  |  | ≥1 | 76 | 32.8 | 26 | 25.6 | 50 | 41.0 |  |
|  |  | 1 | 60 | 25.9 | 23 | 20.9 | 37 | 30.3 |  |
|  |  | 2 | 16 | 6.9 | 3 | 2.7 | 13 | 10.7 |  |
