# Supplementary material for: Structural tissue damage and 24-month progression of semi-quantitative MRI biomarkers of knee osteoarthritis in the IMI-APPROACH cohort
Source: BMC Musculoskelet Disord. 2022 Nov 17;23:988. doi: 10.1186/s12891-022-05926-1 (PMC9670371; doi:10.1186/s12891-022-05926-1)
Supplement: Supplementary file 8 — Additional file 8. [file 12891_2022_5926_MOESM8_ESM.docx]

**Appendix 8.** Improvement in BMLs from baseline to 24 months follow-up

| Number of regions with improvement (**without** within-grade changes, worsening=no-change); N=232 | | | | | | | | | |
| --- | --- | --- | --- | --- | --- | --- | --- | --- | --- |
|  |  |  | All knees | | No ROA | | ROA | | P-value |
|  |  |  | Frequency | Percent | Frequency | Percent | Frequency | Percent |  |
| Knee | Number of regions | 0 | 118 | 50.9 | 75 | 68.2 | 43 | 35.2 | 0.0000 |
|  |  | ≥1 | 114 | 49.1 | 35 | 31.8 | 79 | 64.8 |  |
|  |  | 1 | 62 | 26.7 | 21 | 19.1 | 41 | 33.6 |  |
|  |  | 2 | 37 | 15.9 | 9 | 8.2 | 28 | 23.0 |  |
|  |  | 3 | 10 | 4.3 | 4 | 3.6 | 6 | 4.9 |  |
|  |  | 4 | 4 | 1.7 | 1 | 0.9 | 3 | 2.5 |  |
|  |  | 5 | 1 | 0.4 | 0 | 0.0 | 1 | 0.8 |  |
| MFTJ | Number of regions | 0 | 191 | 82.3 | 101 | 91.8 | 90 | 73.8 | 0.0004 |
|  |  | ≥1 | 41 | 17.7 | 9 | 8.2 | 32 | 26.2 |  |
|  |  | 1 | 32 | 13.8 | 6 | 5.5 | 26 | 21.3 |  |
|  |  | 2 | 8 | 3.4 | 3 | 2.7 | 5 | 4.1 |  |
|  |  | 3 | 1 | 0.4 | 0 | 0.0 | 1 | 0.8 |  |
| LFTJ | Number of regions | 0 | 197 | 84.9 | 105 | 95.5 | 92 | 75.4 | 0.0000 |
|  |  | ≥1 | 35 | 15.1 | 5 | 4.5 | 30 | 24.6 |  |
|  |  | 1 | 25 | 10.8 | 5 | 4.5 | 20 | 16.4 |  |
|  |  | 2 | 6 | 2.6 | 0 | 0.0 | 6 | 4.9 |  |
|  |  | 3 | 3 | 1.3 | 0 | 0.0 | 3 | 2.5 |  |
|  |  | 4 | 1 | 0.4 | 0 | 0.0 | 1 | 0.8 |  |
| PFJ | Number of regions | 0 | 164 | 70.7 | 80 | 72.7 | 84 | 68.9 | 0.4989 |
|  |  | ≥1 | 68 | 29.3 | 30 | 27.3 | 38 | 31.1 |  |
|  |  | 1 | 53 | 22.8 | 24 | 21.8 | 29 | 23.8 |  |
|  |  | 2 | 12 | 5.2 | 4 | 3.6 | 8 | 6.6 |  |
|  |  | 3 | 3 | 1.3 | 2 | 1.8 | 1 | 0.8 |  |
| Number of regions with improvement (**including** within-grade changes, worsening=no change): | | | | | | | | | |
| Knee | Number of regions | 0 | 95 | 40.9 | 67 | 60.9 | 28 | 23.0 | 0.0000 |
|  |  | ≥1 | 137 | 59.1 | 43 | 39.1 | 94 | 77.0 |  |
|  |  | 1 | 62 | 26.7 | 26 | 23.6 | 36 | 29.5 |  |
|  |  | 2 | 48 | 20.7 | 12 | 10.9 | 36 | 29.5 |  |
|  |  | 3 | 15 | 6.5 | 3 | 2.7 | 12 | 9.8 |  |
|  |  | 4 | 9 | 3.9 | 1 | 0.9 | 8 | 6.6 |  |
|  |  | 5 | 3 | 1.3 | 1 | 0.9 | 2 | 1.6 |  |
| MFTJ | Number of regions | 0 | 182 | 78.4 | 100 | 90.9 | 82 | 67.2 | 0.0000 |
|  |  | ≥1 | 50 | 21.6 | 10 | 9.1 | 40 | 32.8 |  |
|  |  | 1 | 32 | 13.8 | 6 | 5.5 | 26 | 21.3 |  |
|  |  | 2 | 14 | 6.0 | 4 | 3.6 | 10 | 8.2 |  |
|  |  | 3 | 4 | 1.7 | 0 | 0.0 | 4 | 3.3 |  |
| LFTJ | Number of regions | 0 | 192 | 82.8 | 104 | 94.5 | 88 | 72.1 | 0.0000 |
|  |  | ≥1 | 40 | 17.2 | 6 | 5.5 | 34 | 27.9 |  |
|  |  | 1 | 26 | 11.2 | 5 | 4.5 | 21 | 17.2 |  |
|  |  | 2 | 9 | 3.9 | 1 | 0.9 | 8 | 6.6 |  |
|  |  | 3 | 4 | 1.7 | 0 | 0.0 | 4 | 3.3 |  |
|  |  | 4 | 1 | 0.4 | 0 | 0.0 | 1 | 0.8 |  |
| PFJ | Number of regions | 0 | 136 | 58.6 | 73 | 66.4 | 63 | 51.6 | 0.0227 |
|  |  | ≥1 | 96 | 41.4 | 37 | 33.6 | 59 | 48.4 |  |
|  |  | 1 | 73 | 31.5 | 29 | 26.4 | 44 | 36.1 |  |
|  |  | 2 | 20 | 8.6 | 6 | 5.5 | 14 | 11.5 |  |
|  |  | 3 | 3 | 1.3 | 2 | 1.8 | 1 | 0.8 |  |
