# Supplementary material for: Structural tissue damage and 24-month progression of semi-quantitative MRI biomarkers of knee osteoarthritis in the IMI-APPROACH cohort
Source: BMC Musculoskelet Disord. 2022 Nov 17;23:988. doi: 10.1186/s12891-022-05926-1 (PMC9670371; doi:10.1186/s12891-022-05926-1)
Supplement: Supplementary file 9 — Additional file 9. [file 12891_2022_5926_MOESM9_ESM.docx]

**Appendix 9.** Osteophyte change (baseline to 24 months)

| N=230 | | | All knees | | No ROA | | ROA | | P |
| --- | --- | --- | --- | --- | --- | --- | --- | --- | --- |
|  |  |  | Frequency | Percent | Frequency | Percent | Frequency | Percent |  |
| Increase in number of locations by category | | | | | | | | | |
| Knee |  | No | 184 | 80.0 | 101 | 92.7 | 83 | 68.6 | 0.0000 |
|  |  | Yes | 46 | 20.0 | 8 | 7.3 | 38 | 31.4 |  |
| MFTJ |  | No | 203 | 88.3 | 103 | 94.5 | 100 | 82.6 | 0.0054 |
|  |  | Yes | 27 | 11.7 | 6 | 5.5 | 21 | 17.4 |  |
| LFTJ |  | No | 208 | 90.4 | 105 | 96.3 | 103 | 85.1 | 0.0040 |
|  |  | Yes | 22 | 9.6 | 4 | 3.7 | 18 | 14.9 |  |
| PFJ |  | No | 216 | 93.9 | 106 | 97.2 | 110 | 90.9 | 0.0452 |
|  |  | Yes | 14 | 6.1 | 3 | 2.8 | 11 | 9.1 |  |
| Increase in number of locations by grade | | | | | | | | | |
| Knee |  | 0 | 184 | 80.0 | 101 | 92.7 | 83 | 68.6 | 0.0000 |
|  |  | 1 | 28 | 12.2 | 5 | 4.6 | 23 | 19.0 |  |
|  |  | 2 | 10 | 4.3 | 1 | 0.9 | 9 | 7.4 |  |
|  |  | 3 | 4 | 1.7 | 0 | 0.0 | 4 | 3.3 |  |
|  |  | 4 | 2 | 0.9 | 1 | 0.9 | 1 | 0.8 |  |
|  |  | 6 | 1 | 0.4 | 1 | 0.9 | 0 | 0.0 |  |
|  |  | 7 | 1 | 0.4 | 0 | 0.0 | 1 | 0.8 |  |
| MFTJ |  | 0 | 203 | 88.3 | 103 | 94.5 | 100 | 82.6 | 0.0053 |
|  |  | 1 | 21 | 9.1 | 5 | 4.6 | 16 | 13.2 |  |
|  |  | 2 | 3 | 1.3 | 0 | 0.0 | 3 | 2.5 |  |
|  |  | 3 | 3 | 1.3 | 1 | 0.9 | 2 | 1.7 |  |
| LFTJ |  | 0 | 208 | 90.4 | 105 | 96.3 | 103 | 85.1 | 0.0043 |
|  |  | 1 | 15 | 6.5 | 2 | 1.8 | 13 | 10.7 |  |
|  |  | 2 | 5 | 2.2 | 2 | 1.8 | 3 | 2.5 |  |
|  |  | 3 | 2 | 0.9 | 0 | 0.0 | 2 | 1.7 |  |
| PFJ |  | 0 | 216 | 93.9 | 106 | 97.2 | 110 | 90.9 | 0.0452 |
|  |  | 1 | 14 | 6.1 | 3 | 2.8 | 11 | 9.1 |  |
| Change in maximum osteophyte score (delta) | | | | | | | | | |
| Knee |  | 0 | 184 | 80.0 | 101 | 92.7 | 83 | 68.6 | 0.0000 |
|  |  | 1 | 44 | 19.1 | 7 | 6.4 | 37 | 30.6 |  |
|  |  | 2 | 2 | 0.9 | 1 | 0.9 | 1 | 0.8 |  |
| MFTJ |  | -1 | 1 | 0.4 | 0 | 0.0 | 1 | 0.8 | 0.0117 |
|  |  | 0 | 202 | 87.8 | 103 | 94.5 | 99 | 81.8 |  |
|  |  | 1 | 26 | 11.3 | 5 | 4.6 | 21 | 17.4 |  |
|  |  | 2 | 1 | 0.4 | 1 | 0.9 | 0 | 0.0 |  |
| LFTJ |  | 0 | 208 | 90.4 | 105 | 96.3 | 103 | 85.1 | 0.0040 |
|  |  | 1 | 22 | 9.6 | 4 | 3.7 | 18 | 14.9 |  |
| PFJ |  | 0 | 216 | 93.9 | 106 | 97.2 | 110 | 90.9 | 0.0444 |
|  |  | 1 | 13 | 5.7 | 3 | 2.8 | 10 | 8.3 |  |
|  |  | 2 | 1 | 0.4 | 0 | 0.0 | 1 | 0.8 |  |
| Any change in maximum score | | | | | | | | | |
| Knee |  | No | 184 | 80.0 | 101 | 92.7 | 83 | 68.6 | 0.0000 |
|  |  | Yes | 46 | 20.0 | 8 | 7.3 | 38 | 31.4 |  |
| MFTJ |  | No | 203 | 88.3 | 103 | 94.5 | 100 | 82.6 | 0.0054 |
|  |  | Yes | 27 | 11.7 | 6 | 5.5 | 21 | 17.4 |  |
| LFTJ |  | No | 208 | 90.4 | 105 | 96.3 | 103 | 85.1 | 0.0040 |
|  |  | Yes | 22 | 9.6 | 4 | 3.7 | 18 | 14.9 |  |
| PFJ |  | No | 216 | 93.9 | 106 | 97.2 | 110 | 90.9 | 0.0452 |
|  |  | Yes | 14 | 6.1 | 3 | 2.8 | 11 | 9.1 |  |
