# Supplementary material for: Structural tissue damage and 24-month progression of semi-quantitative MRI biomarkers of knee osteoarthritis in the IMI-APPROACH cohort
Source: BMC Musculoskelet Disord. 2022 Nov 17;23:988. doi: 10.1186/s12891-022-05926-1 (PMC9670371; doi:10.1186/s12891-022-05926-1)
Supplement: Supplementary file 10 — Additional file 10. [file 12891_2022_5926_MOESM10_ESM.docx]

**Appendix 10.** Meniscus change baseline to 24 months

| N=221 | | | All knees | | No ROA | | ROA | | P-value |
| --- | --- | --- | --- | --- | --- | --- | --- | --- | --- |
|  |  |  | Frequency | Percent | Frequency | Percent | Frequency | Percent |  |
| Change in number of regions with any change in meniscus morphology | | | | | | | | | |
| Medial | Number of regions | 0 | 206 | 93.2 | 101 | 94.4 | 105 | 92.1 | 0.4914 |
|  |  | 1 | 9 | 4.1 | 4 | 3.7 | 5 | 4.4 |  |
|  |  | 2 | 6 | 2.7 | 2 | 1.9 | 4 | 3.5 |  |
| Lateral | Number of regions | 0 | 206 | 93.2 | 104 | 97.2 | 102 | 89.5 | 0.0220 |
|  |  | 1 | 13 | 5.9 | 3 | 2.8 | 10 | 8.8 |  |
|  |  | 2 | 2 | 0.9 | 0 | 0.0 | 2 | 1.8 |  |
| Change in number of regions with any categorical change (no to tear, tear to maceration) in meniscus morphology | | | | | | | | | |
| Medial | Number of regions | 0 | 212 | 95.9 | 102 | 95.3 | 110 | 96.5 | 0.6647 |
|  |  | 1 | 7 | 3.2 | 4 | 3.7 | 3 | 2.6 |  |
|  |  | 2 | 2 | 0.9 | 1 | 0.9 | 1 | 0.9 |  |
| Lateral | Number of regions | 0 | 212 | 95.9 | 105 | 98.1 | 107 | 93.9 | 0.1066 |
|  |  | 1 | 7 | 3.2 | 2 | 1.9 | 5 | 4.4 |  |
|  |  | 2 | 2 | 0.9 | 0 | 0.0 | 2 | 1.8 |  |
| Presence of change in number of regions with any change in meniscus morphology | | | | | | | | | |
| Medial | Any change | No | 206 | 93.2 | 101 | 94.4 | 105 | 92.1 | 0.5003 |
|  |  | Yes | 15 | 6.8 | 6 | 5.6 | 9 | 7.9 |  |
| Lateral | Any change | No | 206 | 93.2 | 104 | 97.2 | 102 | 89.5 | 0.0229 |
|  |  | Yes | 15 | 6.8 | 3 | 2.8 | 12 | 10.5 |  |
| Change in root tears | | | | | | | | | |
| Medial | Root tear | No | 219 | 99.1 | 107 | 100.0 | 112 | 98.2 | 0.1697 |
|  |  | Yes | 2 | 0.9 | 0 | 0.0 | 2 | 1.8 |  |
| Lateral | Root tear | No | 220 | 99.5 | 107 | 100.0 | 113 | 99.1 | 0.3326 |
|  |  | Yes | 1 | 0.5 | 0 | 0.0 | 1 | 0.9 |  |
| Knees with increase in extrusion | | | | | | | | | |
| Medial | Grade (delta) | 0 | 197 | 89.1 | 96 | 89.7 | 101 | 88.6 | 0.7461 |
|  |  | 1 | 17 | 7.7 | 9 | 8.4 | 8 | 7.0 |  |
|  |  | 2 | 7 | 3.2 | 2 | 1.9 | 5 | 4.4 |  |
| Lateral | Grade (delta) | 0 | 216 | 97.7 | 106 | 99.1 | 110 | 96.5 | 0.1994 |
|  |  | 1 | 5 | 2.3 | 1 | 0.9 | 4 | 3.5 |  |
| Presence of increase of extrusion | | | | | | | | | |
| Medial | Any change | No | 197 | 89.1 | 96 | 89.7 | 101 | 88.6 | 0.7890 |
|  |  | Yes | 24 | 10.9 | 11 | 10.3 | 13 | 11.4 |  |
| Lateral | Any change | No | 216 | 97.7 | 106 | 99.1 | 110 | 96.5 | 0.1994 |
|  |  | Yes | 5 | 2.3 | 1 | 0.9 | 4 | 3.5 |  |
